# NC Meets CN: Porous Photoanodes with Polymeric Carbon Nitride/ZnSe Nanocrystal Heterojunctions for Photoelectrochemical Applications

**DOI:** 10.1021/acsami.4c07582

**Published:** 2024-07-16

**Authors:** Sanjit Mondal, Tom Naor, Michael Volokh, David Stone, Josep Albero, Adar Levi, Atzmon Vakahi, Hermenegildo García, Uri Banin, Menny Shalom

**Affiliations:** † Department of Chemistry and Ilse Katz Institute for Nanoscale Science and Technology, Ben-Gurion University of the Negev, Beer-Sheva 8410501, Israel; ‡ The Institute of Chemistry and The Center for Nanoscience and Nanotechnology, The Hebrew University of Jerusalem, Jerusalem 91904, Israel; § Instituto Universitario de Tecnología Química CSIC-UPV, Universitat Politècnica de València, València 46022, Spain

**Keywords:** carbon nitride, semiconductor nanocrystals, ZnSe, photoelectrochemical cells, electrophoretic deposition

## Abstract

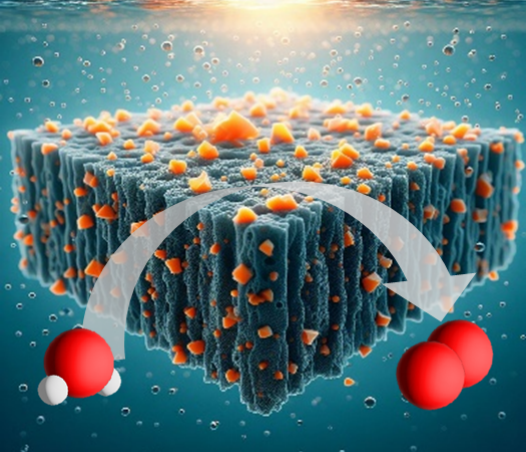

The utilization of photoelectrochemical cells (PEC) for converting solar energy into fuels (e.g., hydrogen) is a promising method for sustainable energy generation. We demonstrate a strategy to enhance the performance of PEC devices by integrating surface-functionalized zinc selenide (ZnSe) semiconductor nanocrystals (NCs) into porous polymeric carbon nitride (CN) matrices to form a uniformly distributed blend of NCs within the CN layer via electrophoretic deposition (EPD). The achieved type II heterojunction at the CN/NC interface exhibits intimate contact between the NCs and the CN backbone since it does not contain insulating binders. This configuration promotes efficient charge separation and suppresses carrier recombination. The reported CN/NC composite structure serves as a photoanode, demonstrating a photocurrent density of 160 ± 8 μA cm^–2^ at 1.23 V vs a reversible hydrogen electrode (RHE), 75% higher compared with a CN-based photoelectrode, for approximately 12 h. Spectral and photoelectrochemical analyses reveal extended photoresponse, reduced charge recombination, and successful charge transfer at the formed heterojunction; these properties result in enhanced PEC oxygen production activity with a Faradaic efficiency of 87%. The methodology allows the integration of high-quality colloidal NCs within porous CN-based photoelectrodes and provides numerous knobs for tuning the functionality of the composite systems, thus showing promise for achieving enhanced solar fuel production using PEC.

## Introduction

Converting solar energy into fuels (e.g., hydrogen) via photoelectrochemical cells (PECs) is a promising route toward sustainable energy supply.^1−5^ At the heart of a PEC device lies a light-harvesting semiconductor (SC), which, under solar illumination, transforms the absorbed photons into excited charge carriers (electrons and holes).^3,6−11^ Such PEC photoelectrodes require cheap and abundant materials, manifesting in efficient solar-to-fuel conversion and high stability. Thus far, research in this field has focused mainly on inorganic SCs such as metal-oxides,^12^ -oxynitrides,^13^ -sulfides,^14^ etc. Improved functionality may be achieved by combining two SC-based photocatalytic water-splitting systems into a nanocomposite, as one can utilize the advantages of each component, preferably in a synergetic manner that exceeds a simple sum of its parts, e.g., by forming a charge separating heterojunction for enhanced PEC efficiency.^15,16^ Following this powerful approach, herein we introduce the combination of a semiconducting polymeric carbon nitride (CN; specifically, with embedded reduced graphene oxide (rGO) as an electron conductor)^17^ with colloidal semiconductor nanocrystals (SC NCs), specifically ZnSe,^18^ into a nanocomposite for PEC, hence the concept of NC meets CN.

Polymeric CNs, the first component in our PEC design, are an emerging family of n-type materials with a prospect of serving as a photoanode active layer since they have suitable energy band positions for water-splitting, excellent chemical and thermal stability, earth-abundance of their constituents, available precursors, and low cost.^19−23^ Significant progress was achieved by developing several approaches for the growth of uniform CN layers. These advancements consist of increased control over several properties of CNs (e.g., extent of condensation, degree of crystallinity, and physical adhesion to the substrate) and control over the structure and composition of the CN layer, including the formation of homojunctions, the incorporation of conductive rGO, heteroatoms, or carbon incorporation.^19,24−28^ However, CN’s activity is limited by inherent poor exciton separation and nonoptimal light absorption (optical band gap, *E*_g_ ∼ 2.7 eV). Thus, modifications of the CN layer, its blending with conductive additives, or interfacing with homojunctions or heterojunctions are among the sought-after strategies to achieve efficient CN-based photoanodes.^29−35^

SC NCs are the second component of our approach, which, along with the inherent high surface-to-volume ratio essential for efficient photocatalysis,^36^ stand out in the ability to control their properties via adjustment of particle size, shape, and composition.^37,38^ This enables the tuning of their optical absorption to match the relevant parts of the solar spectrum and the adjustment of the band positions to meet the requirements of the photocatalytic redox reactions. Furthermore, the chemical flexibility enabled by manipulation of the NC’s surface chemistry opens up opportunities for their incorporation in diverse solvents and matrices via straightforward and affordable solution-based methods. Indeed, such NCs, along with grown metal cocatalysts, have been demonstrated as efficient free-standing photocatalysts for hydrogen generation via water-splitting while utilizing sacrificial hole acceptors.^39−43^ However, applying SC NCs in PEC lags behind due to the difficulty in forming a continuous, conducting photocatalytic NC layer. Moreover, cadmium-based NCs are leading the field due to the ease and flexibility of the synthesis, stability in aqueous solutions, and favorable balance between solar spectrum absorption and the required overpotential for water reduction, but despite their advantages, environmental concerns surrounding cadmium necessitate the development of cadmium-free systems.^41,44,45^ In this context, ZnSe NCs are an excellent case-study with an appropriate band gap of 2.7 eV in the visible range.^18,46−49^

Despite the significant progress in the individual utilization of CN and SC NCs in photocatalysis,^39,50,51^ their activity is limited by their properties and the synthetic difficulties in the growth and deposition of the photoactive materials while (i) forming intimate contact with the conductive substrate and (ii) maintaining a porous structure with sufficient conductivity within the layer to allow efficient charge carrier separation, as well as yielding the desired reaction on the surface. Optimal functionality of the composite NC–CN PEC device requires homogeneous coverage of the base material from its top exposed layers all the way to the bottom substrate-contacted layers. To achieve this, we utilize electrophoretic deposition (EPD), which has emerged as a powerful and versatile method for the controlled assembly of NCs onto various substrates for numerous applications. This simple and scalable process allows for the formation of a uniform NC coating by leveraging the principles of electrophoresis to deposit charged NCs within a colloidal suspension onto conducting substrates via the application of an electric field.^52−55^

In this work, we report a general strategy to combine NCs with porous CN systems via EPD to form functional photoelectrodes composed of a uniformly distributed blend of NCs within the CN layer. The CN/NC interface should allow intimate contact without using insulating binders, maintaining stable contact between the constituents as well as between the layer and the transparent conductive substrate, acting as the illuminated back-contact of the photoanode in a typical PEC configuration. The merits of the described structure include superior exciton separation, suppressing charge carrier recombination, extension of the harvested spectrum, and enhancement of the available active sites’ density.^56^ Specifically, using EPD, we interface a porous CN with blended reduced graphene oxide (rGO) serving as an electron conductor^17^ (i.e., the CNGO matrix) with suitably surface-functionalized ZnSe NCs. This unique combination in a single nanocomposite photoanode results in a water-splitting PEC device exhibiting a photocurrent density of 160 ± 8 μA cm^–2^ at 1.23 V vs a reversible hydrogen electrode (RHE) in an alkaline medium, 75% higher compared with the pristine CNGO photoelectrode. Spectral and photoelectrochemical measurements reveal that the formed heterojunction manifests a charge-separating staggering band offset, which exhibits an extended photoresponse, lower charge recombination, and successful charge transfer. Importantly, the NC–CN combination significantly improves the photoanode’s stability toward cocatalyst-free PEC.

## Results and Discussion

**Scheme 1:**
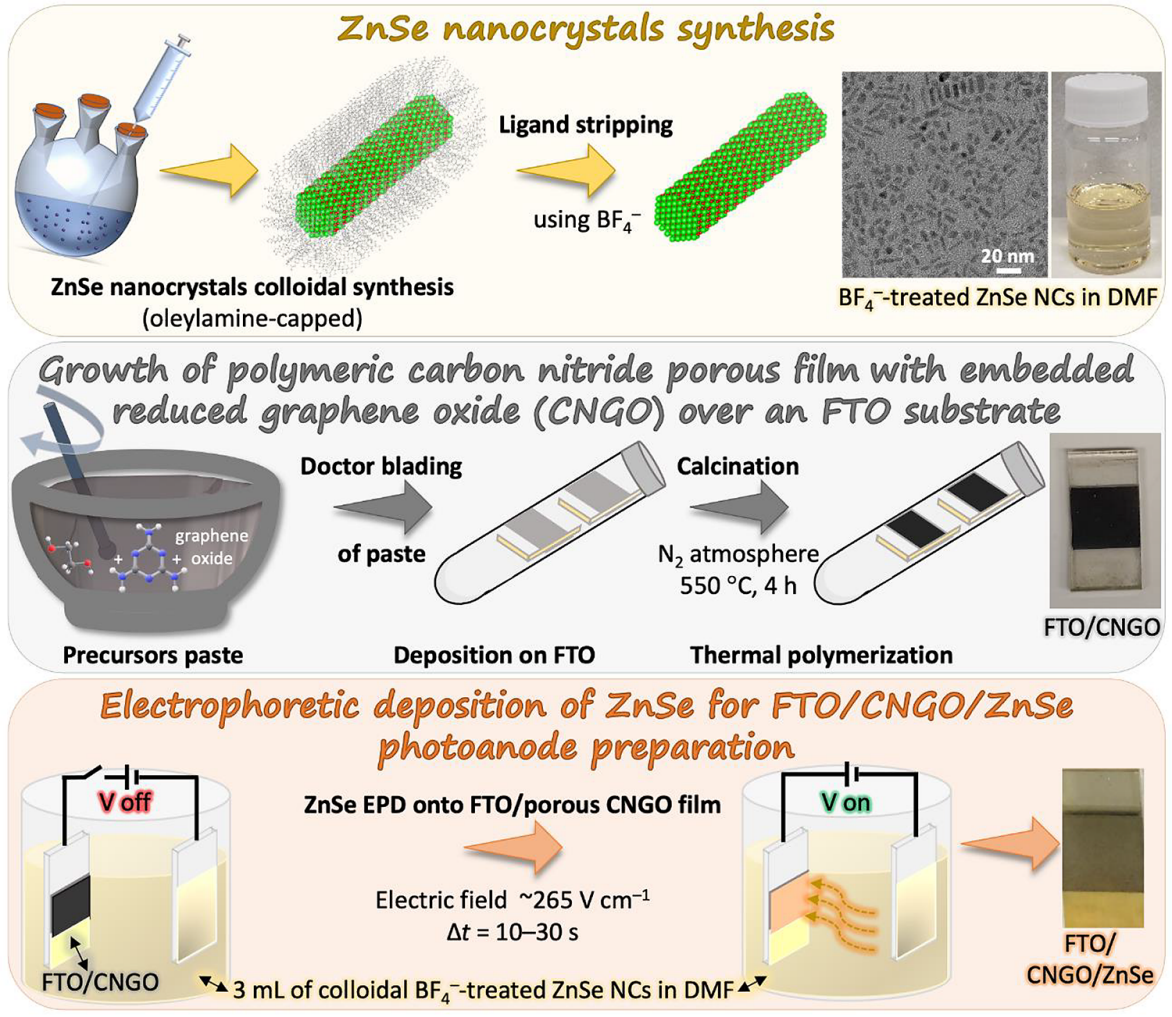
Synthetic Steps Used to Form a Photoanode Based on a CNGO/ZnSe Heterostructure Film Over FTO Synthesis of ZnSe nanocrystals and ligand exchange; growth of a porous polymeric carbon nitride film over FTO with embedded reduced graphene oxide (CNGO); electrophoretic deposition of ZnSe nanocrystals onto the CNGO film resulting in a FTO/CNGO/ZnSe photoanode.

The nanocomposite “NC meets CN” photoelectrode was fabricated in two parallel routes (Scheme 1): ZnSe NCs synthesis and surface modification and CNGO film growth over an FTO-coated glass. The two components were then combined into one nanocomposite system by using EPD of the ZnSe NCs onto the porous polymeric CN film.

ZnSe NCs were synthesized based on a previously reported synthesis,^57^ using Zn stearate and selenourea as the zinc and selenium precursors, respectively, and oleylamine (OAm) as a coordinating solvent, with a small amount of dodecanethiol. Briefly, after the synthesis was heated to 170 °C under an inert environment, ZnSe nanowires were obtained through oriented attachment growth from small clusters. Then, upon heating to 250 °C, a ripening process that shortens and thickens the wires led to the formation of rod- and dome-shaped NCs. The ZnSe NC size and shape were determined using transmission electron microscopy (TEM) imaging (Figure S1a). Two main morphologies were observed: nanorods (15.5 ± 3.6 nm long, 3.8 ± 0.8 nm in diameter) and dome-shaped NCs (8.1 ± 1.1 nm base diameter, 5.9 ± 0.9 nm height). A ratio of 3:1 nanodomes to nanorods was calculated from the TEM images. The hexagonal wurtzite ZnSe crystal structure was determined based on a high-resolution TEM (HRTEM) image, analyzed by fast Fourier transform (FFT) (Figure S1b and inset). When viewing down the [0002] zone axis, the (0002) and (112̅0) dominant facets are noticeable, corresponding to their *d*-spacing and angles.^58^

The as-synthesized ZnSe NCs are passivated with OAm, which can hinder the charge transfer processes among the NCs and between the NCs and the CN matrix.^59^ Addressing this, the NCs underwent a ligand-stripping procedure with BF_4_^–^, which also facilitated the dispersion of the post-treatment, positively charged ZnSe NCs in polar solvents (DMF),^59^ beneficial for the EPD process. The outcome of the ligand stripping process is clearly resolved via thermal gravimetric analysis (TGA) for the ZnSe NC solution before and after surface treatment (Figure S1c, OAm and BF_4_^–^, respectively). The initial OAm-coated sample showed a weight loss of ∼30% at 400 °C, consistent with a monolayer of the OAm organic ligands coating the NC surface (see Note S1 in the Supporting Information). After the reaction with BF_4_^–^, only a slight material loss was observed, indicating the efficient stripping of the OAm ligands from the NCs’ surface. Furthermore, there was no change in the absorption spectrum of ZnSe NCs, with an absorption onset at 450 nm before and after the surface treatment (Figure S1d). XRD diffraction patterns, before and after the ligand stripping process, show no significant change in peak positions (Figure S1e). We attribute the different intensity ratios between the peaks to the preferable orientation of the NCs during EPD over FTO.

In order to fabricate the NC–CN composite photoanode, we employed the EPD method to deposit the ligand-stripped ZnSe NCs onto the porous CNGO matrix. A solution of the ZnSe NCs in DMF (∼4 μM concentration) was used in a parallel capacitor configuration between two FTO substrates, and 80 V was applied for 5, 10, or 15 s. Deposition of the positively charged ZnSe NCs occurred on the CNGO-coated FTO negatively biased electrode, as indicated visually by the change of color from black to a gray-yellow tone (photos in Scheme 1), yielding an FTO/CNGO/ZnSe electrode.

**Figure 1:**
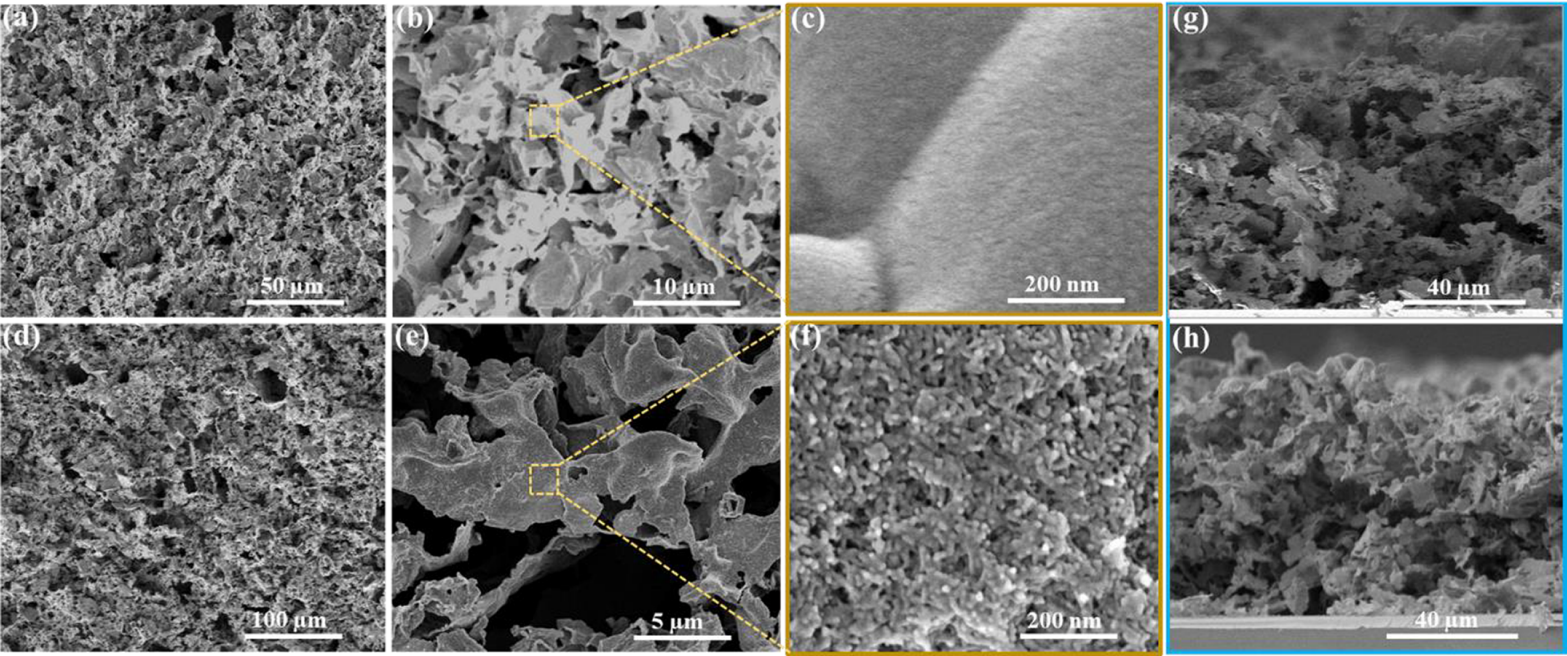
(a–c) SEM images (top-view) of the CNGO. (d–f) Top-view SEM images of CNGO/ZnSe (10 s) film. (g,h) Cross-sectional SEM image of (g) CNGO and (h) CNGO/ZnSe (10 s) film, respectively.

The films were imaged by using scanning electron microscopy (SEM) to analyze the details of the NC deposition. The pristine CNGO electrode revealed a porous, thin sheet-like structure of the CN (Figure 1a,b). The sheets are interconnected, and the evident pores are in the micrometer range. This morphology is retained after the EPD process in the final structure of the CNGO/ZnSe films (Figure 1d,e). A high-magnification SEM image of the pristine CNGO reveals the smooth surface of the sheets (Figure 1c), while for the CNGO/ZnSe, the CN surface is covered with rod-shaped ZnSe NCs throughout the film (Figure 1f). A cross-sectional view reveals a porous uniform CN layer with a thickness between 90–100 μm for the CNGO electrode that remains intact for the CNGO/ZnSe film (Figure 1g,h).

**Figure 2:**
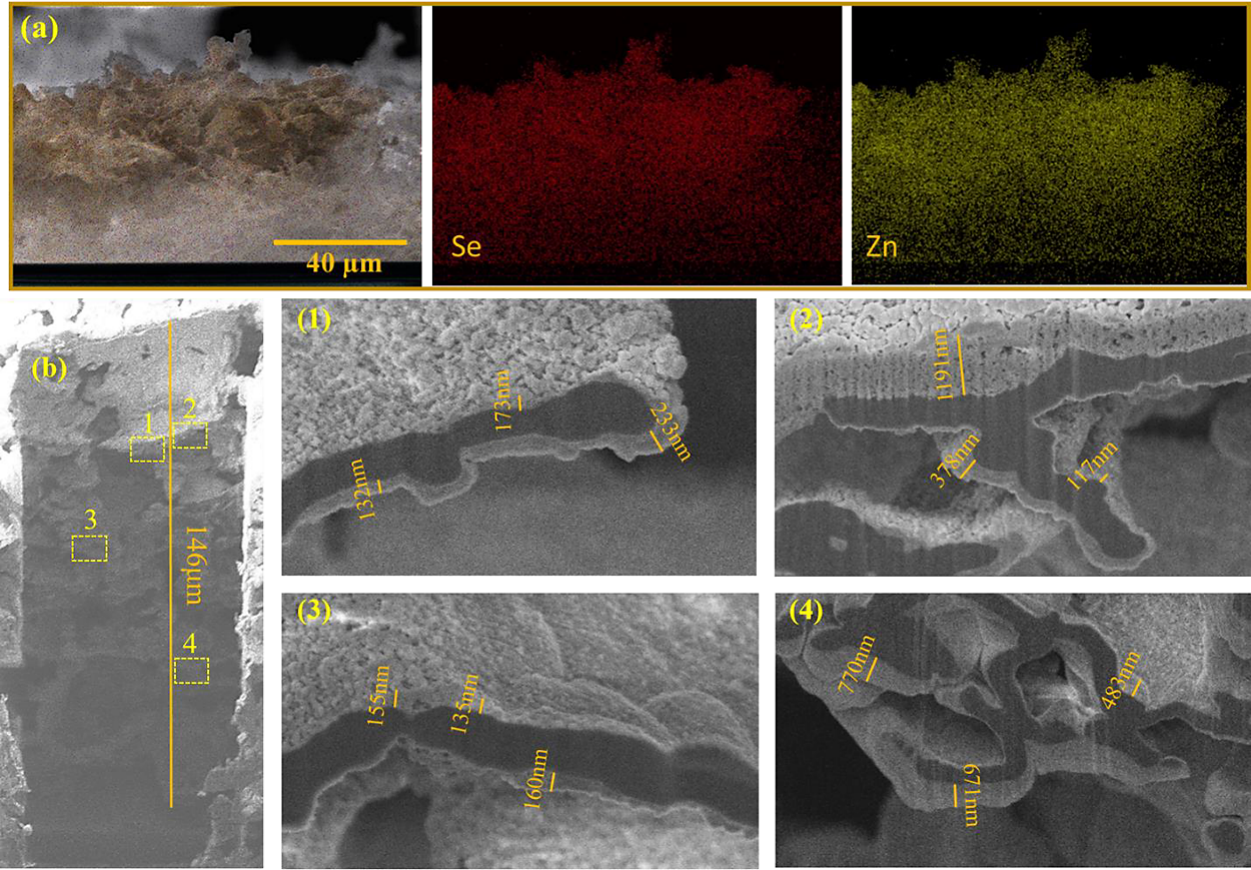
(a) EDS–mapping of CNGO/ZnSe film in the cross-sectional area (Se and Zn). (b) FIB–SEM image of CNGO/ZnSe film in the cross-sectional area at different depths (1,2: ∼40 μm; 3: ∼70 μm; and 4: ∼110 μm) of the film.

Energy-dispersive X-ray spectroscopy (EDS) shows the elemental mapping of the CNGO/ZnSe film’s cross-sectional area. Figure 2a demonstrates that the ZnSe NCs are uniformly distributed throughout the film, all the way to the FTO surface. This result is further supported by the depth profile achieved with focused ion beam (FIB) SEM imaging (Figure 2b), which shows the complete coverage of the porous CNGO film (dark) by a 100–1000 nm layer of ZnSe NCs (bright). The EPD method thus ensures a relatively uniform distribution of NCs across the film, facilitated by the porous structure of the CN matrix.

The crystallinity and structure of the ZnSe NCs and CNGO electrodes, before and after EPD of the ZnSe NCs, were further investigated by using X-ray diffraction (XRD) analysis (Figure S2a). The XRD pattern of ZnSe NCs deposited by EPD directly on FTO showed main diffraction signals at 26°, 27.4°, 45.4°, and 53.8°, attributed to the (101̅0), (0002), (112̅0), and (112̅2) planes of the ZnSe hexagonal wurtzite structure, respectively. The diffraction at 26° is of lower intensity, but the other three peaks are also noticed for the CNGO/ZnSe combined films. A strong and sharp reflection observed at 27.4° in the XRD pattern of the CNGO electrodes is attributed to the (002) interlayer distance of the CN. X-ray photoelectron spectroscopy (XPS) analysis was also performed, and following the Zn (at 1044.6, 1021 eV) and Se (at 54.2, 53.3 eV) peaks clearly further validates the presence of ZnSe NCs at the surface of the CNGO/ZnSe electrode (Figure S2b).^60^ Moreover, the C 1s and N 1s XPS spectra of both the CNGO and CNGO/ZnSe films were analyzed. The C 1s spectrum of CNGO consists of two peaks at 284.8 and 288.3 eV, corresponding to adventitious carbon contaminants and sp^2^ hybridized carbon (N–C=N) in the aromatic skeleton rings, respectively (Figure S3), whereas the N 1s XPS spectrum is deconvoluted into three peaks at 398.75, 400.12, and 401.23 eV, attributed to sp^2^-hybridized nitrogen (C–N=C), sp^3^-hybridized nitrogen in a tertiary amine (N–(C)_3_), and sp^3^-hybridized nitrogen in a secondary amine (H–N–(C)_2_), respectively. In contrast, the C 1s spectrum of the CNGO/ZnSe film exhibits three additional peaks at 283.2, 286.1, and 290.3 eV, and the N 1s spectrum contains a single additional peak at 402.5 eV, potentially originating from the ligands present in the ZnSe NCs.

**Figure 3:**
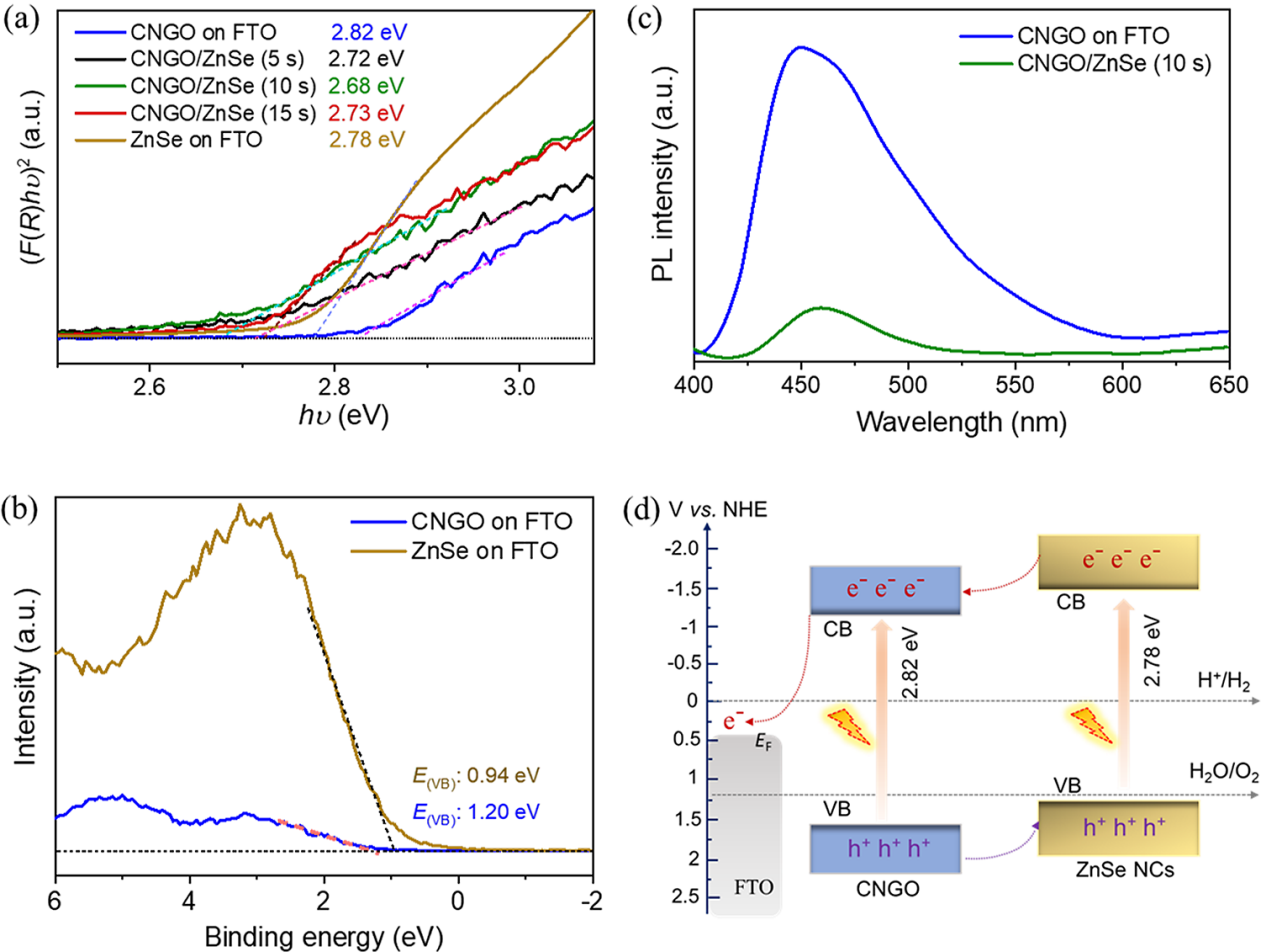
(a) Tauc plot analysis of CNGO, CNGO/ZnSe, and ZnSe NC films assuming a direct band gap (*E*_g_). (b) Valence band (VB) XPS of CNGO and ZnSe electrodes. (c) PL emission spectra of the CNGO and CNGO/ZnSe films. (d) Schematic representation of the electronic band structure of CNGO and ZnSe vs NHE, determined using the XPS-measured VB position and the optical *E*_g_ estimation, showing the type-II heterojunction (staggered energy levels).

Fourier-transform infrared (FTIR) spectra of both CNGO and CNGO/ZnSe films exhibited similar peaks in the 1180–1630 cm^–1^ range, which are assigned to the characteristic stretch modes of aromatic CN heterocycles (Figure S4), whereas the peak at 812 cm^–1^ corresponds to the breathing mode of the heptazine units.^61,62^ The broad band between 3000 and 3600 cm^–1^ is consistent with the presence of −NH_2_ groups. UV–vis diffuse reflectance spectroscopy (DRS) of the CNGO electrode (Figure S5) shows an absorption onset of 450 nm with a direct band gap (*E*_g_) of 2.82 eV, obtained from the Tauc plot as displayed in Figure 3a, while the modified CNGO with ZnSe NCs demonstrates an extended absorption tail beyond 500 nm with an estimated band gap of 2.68 eV.

To estimate the valence band (VB) position of the ZnSe NCs and the CNGO films, VB XPS was performed (Figure 3b).^63^ The calculated VB potentials for pristine ZnSe and CNGO films are 1.34 and 1.61 V vs NHE, respectively, using eq S8 (see Note S2 in the Supporting Information). The steady-state photoluminescence (PL) spectra of CNGO and CNGO/ZnSe electrodes are shown in Figure 3c. The emission intensity of the CNGO decreased after incorporating ZnSe NCs onto the CNGO film due to the type-II band alignment facilitating exciton separation, which decreased the radiative recombination. By combining the optical band gap with the calculated VB potential (obtained from VB XPS analysis), we obtained a proposed energy band diagram of the CNGO/ZnSe NC composite (Figure 3d). The estimated band structure suggests that ZnSe and CNGO form a type-II heterojunction. Accordingly, under illumination, the photoexcited electrons will migrate from the conduction band (CB) of ZnSe to the CB of CNGO; this spatial separation facilitates further migration of electrons until collection at the FTO, while the holes move in the opposite direction (able to perform an oxidation reaction at the NC/electrolyte interface).

We conducted spectroscopic analyses to understand the carrier concentration, charge transfer resistance, and photoinduced charge transfer kinetics in NC–CN films. The charge carrier density (*N*_D_) of the CNGO and CNGO/ZnSe films was calculated via Mott–Schottky analysis. Figure S6 reveals that *N*_D_ is notably higher in the CNGO/ZnSe film (3.6 × 10^18^ cm^–3^) compared to a CNGO film (1.5 × 10^17^ cm^–3^), which should incite a faster charge carrier transfer process than that in CNGO alone, and thus the composite film might exhibit better PEC performance.^64^ Electrochemical impedance spectroscopy (EIS) at different potentials (0.05, 0.1, 0.15, and 0.25 V vs Ag/AgCl) showed reduced charge transfer resistance in CNGO/ZnSe compared to CNGO, indicating improved charge transfer, which we attribute to the spatial separation of excited charge carriers across the heterojunction as a result of the type–II band alignment (Figure S7).^65,66^ Chronopotentiometric open circuit potential (*V*_OC_) measurements for CNGO and CNGO/ZnSe films further examine the quasi-equilibrium attained in the dark or under illumination, where generation, accumulation, and recombination of photoinduced electron–hole pairs take place. Compared to a CNGO film alone, the CNGO/ZnSe composite photoanode exhibits a more negative *V*_OC_ under 1 sun illumination (Figure S8), suggesting that the presence of ZnSe NCs facilitates the accumulation of photoinduced electrons within the CB of CNGO, thereby promoting the separation of excitons.

Furthermore, transient absorption spectroscopy (TAS) confirmed enhanced photoinduced charge transfer kinetics with ZnSe NCs. CNGO/ZnSe exhibited distinct TAS spectra with increased optical density, suggesting higher excited state concentration and enhanced charge separation efficiency (Figure S9a; see further discussion in Note S3, Supporting Information). The TA decays at 375 and 500 nm showed two distinct kinetics in CNGO/ZnSe, with longer lifetimes compared to CNGO alone, suggesting slower recombination kinetics (Figure S9b,c and Note S3, Supporting Information). Overall, the formation of the heterojunction not only improved charge transfer kinetics but also slowed down recombination rates, both critical factors for achieving high performance in PEC devices.

**Figure 4:**
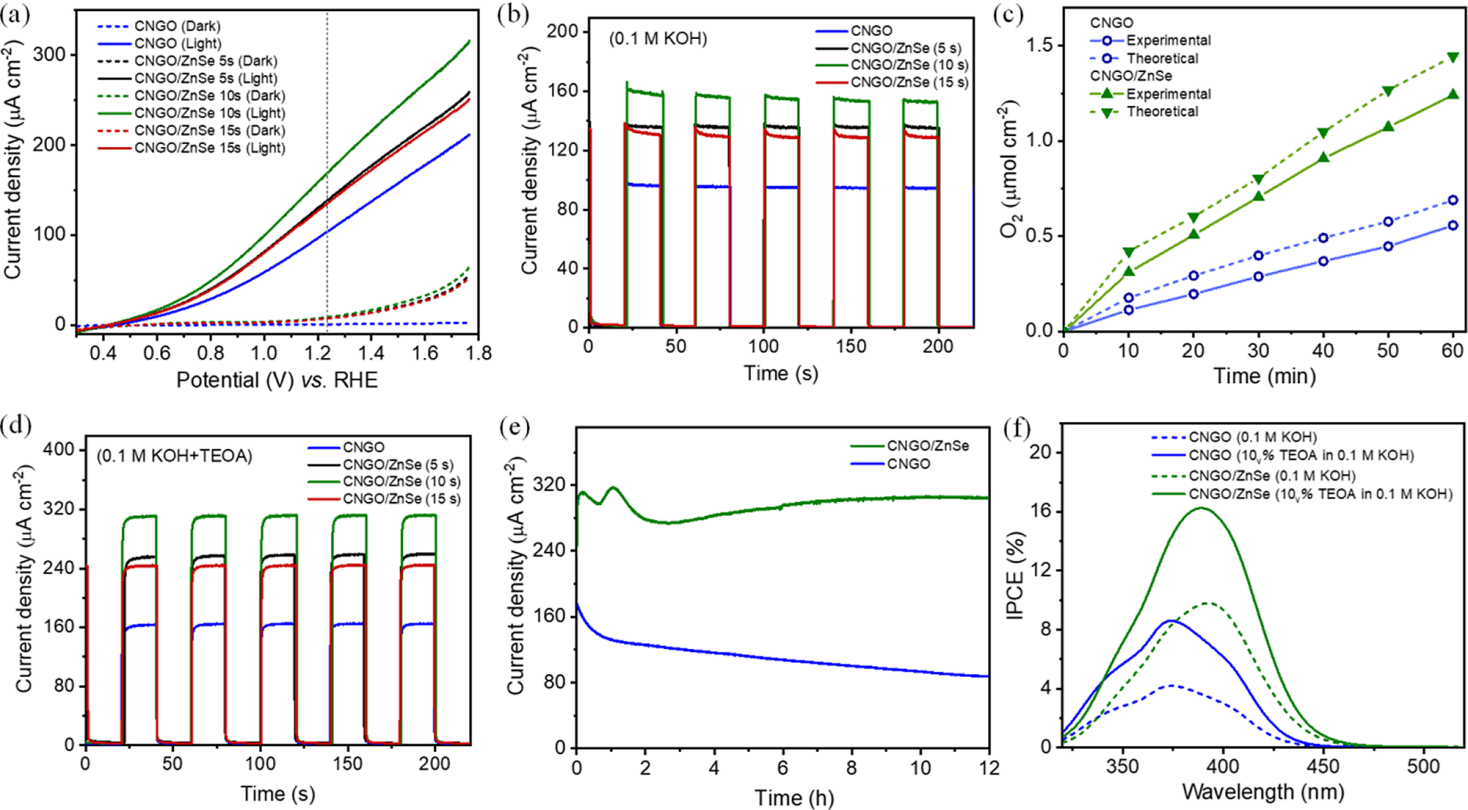
Photoelectrochemical characterization of FTO/CNGO and FTO/CNGO/ZnSe NC photoanodes in a 3-electrode configuration water-splitting undivided single cell (Pt counter electrode serving as the cathode). (a) LSV plots in 0.1 M KOH electrolyte (scan rate, 50 mV s^–1^). (b) Chronoamperometry in 0.1 M KOH at 1.23 V vs RHE. (c) Measured the O_2_ production for CNGO and CNGO/ZnSe in 0.1 M KOH. (d) Chronoamperometry of CNGO and CNGO/ZnSe films in 0.1 M KOH solution containing 10% v/v TEOA hole scavenger at 1.23 V vs RHE. (e) Chronoamperometric stability of CNGO and CNGO/ZnSe (10 s) films in 0.1 M KOH containing 10% v/v TEOA hole scavenger at 1.23 V vs RHE. (f) IPCE measurement of CNGO and CNGO/ZnSe films in 0.1 M KOH and in 0.1 M KOH solution containing 10% v/v TEOA.

The photoelectrochemical functionality of the composite CNGO/ZnSe NC films on FTO was evaluated via comparison of their performance as photoanodes in the oxygen evolution reaction (OER) to pristine CNGO on FTO. This was tested in 0.1 M KOH aqueous solutions using a customized three-electrode cell at 1.23 V vs RHE under 1 sun illumination. Cyclic voltammetry plots of CN, CNGO, and CNGO/ZnSe films in 0.1 M aqueous KOH solution (Figure S10) complements the comparison by showing the enhanced electrochemical response following the blending of rGO^17^ and further improvement after the deposition of ZnSe NCs. Figure 4a presents linear sweep voltammetry (LSV) curves of CNGO and CNGO/ZnSe films prepared with different deposition times (5, 10, and 15 s) in the dark and under illumination, showing a typical PEC behavior with low onset potentials of 0.45 and 0.41 V, respectively, indicating that the photocurrent starts ∼1 V below the “dark” thermodynamic OER potential (1.23 V vs RHE). It was noticed that CNGO/ZnSe (10 s) exhibited a higher current density at 1.23 V vs RHE under illumination compared to other films. Figure S11 compares the applied bias photon-to-current conversion efficiency (ABPE) of CNGO and CNGO/ZnSe films. The maximum ABPE of CNGO/ZnSe is 0.0242%, which is almost double that of CNGO alone (≈0.014%) at 0.92 V vs RHE. The CNGO/ZnSe films prepared with different deposition times (5, 10, 15 s) exhibited significant photoresponse enhancement in comparison with bare CNGO, as shown in the chronoamperometric measurements at 1.23 V vs RHE (Figure 4b). The film prepared with a 10 s deposition time reached the highest stable photocurrent density of 160 ± 8 μA cm^–2^. The enhanced response of the NC–CN composites is attributed to the improved light harvesting and better charge separation in the hybrid CNGO/ZnSe system in line with the type–II heterojunction formation, as well as the lower charge transfer resistance.

Particular focus is given below for the 10 s EPD CNGO/ZnSe system, which showed the highest photocurrent density, suggesting an optimal thickness of the ZnSe conformal coating. Further PEC activity measurements of CNGO/ZnSe in both acidic (0.5 M H_2_SO_4_, pH ∼ 0.3) and neutral (phosphate buffer, pH 7) electrolytes obtained photocurrent densities of 120 ± 8 and 155 ± 8 μA cm^–2^, respectively (Figure S12). The good activity over a wide pH range opens the possibility of using the new photoanode in other oxidation reactions that are pH-sensitive.

Significantly, the O_2_ quantification measurements (Figure 4c) show that the rate of generation of the O_2_ increases substantially from 0.009 μmol cm^–2^ min^–1^ for CNGO to 0.020 μmol cm^–2^ min^–1^ for CNGO/ZnSe. Analyzing the theoretical expected O_2_ formation from the photocurrent data shows a high overall Faradaic efficiency (FE) of 68% for the CNGO, which impressively increases to 87% for CNGO/ZnSe at 30 min during the hour-long water-splitting PEC experiment, meaning that most of the holes are consumed for water oxidation and do not participate in parasitic self-oxidation of the active layer (Figure S13). Moreover, H_2_ quantification (Figure S14) reveals hydrogen evolution rates of 0.017 μmol cm^–2^ min^–1^ for the CNGO film and 0.040 μmol cm^–2^ min^–1^ for the CNGO/ZnSe film. At 40 min, the CNGO/ZnSe films show a H_2_ FE of 88% (compared to 70% for CNGO only films), demonstrating that close to 90% of the photogenerated charge carriers in this time frame successfully separate, reach the relevant electrode, and perform the redox reaction (holes reach the CNGO/ZnSe photoanode/electrolyte interface as discussed before, and importantly, electrons migrate through the composite photoanode and reach the Pt counter electrode, where they perform the reduction reaction).

To further address the mechanism and the limiting factors in the PEC performance, photocurrent densities were measured in the presence of a hole scavenger, triethanolamine (TEOA; 10% v/v), in a 0.1 M KOH aqueous solution at 1.23 V vs RHE (Figures 4d and S16). Three sets of measurements were performed for CNGO and CNGO/ZnSe photoanodes with and without the addition of TEOA (Figure S15), allowing the calculation of a standard deviation. In the presence of TEOA, elevated values of 160 ± 8 and 318 ± 8 μA cm^–2^ were measured for bare CNGO and CNGO/ZnSe electrodes, respectively, representing an increase (by 78 and 100%, respectively) relative to the case without hole scavenger. We conclude from this that a significant limiting factor in both cases is hole extraction at the photoanode/electrolyte interface. In particular, in the composite CNGO/ZnSe active film, which comprises type-II heterojunctions, the somewhat higher increase in the presence of a hole scavenger is consistent with the improved hole charge transfer characteristics and suppressed electron–hole recombination. This is further supported by the quenching of PL upon ZnSe NC deposition (Figure 3c).

The relevance of hole extraction for improving the CNGO/ZnSe composite film is further born out from the assessment of the stability of the films in the presence and absence of the hole acceptor TEOA. This unveiled a remarkable enhancement in stability due to the incorporation of ZnSe NCs over a 12 h period (Figure 4e vs Figure S17a). The CNGO/ZnSe photoanode retains close to 100% activity even after a continuous 12 h operation in 0.1 M KOH containing 10% v/v TEOA hole scavenger at 1.23 V vs RHE, which can be attributed to the enhanced charge separation originating from the type–II heterojunction formed between the materials. This high stability opens the possibility for other organic molecules’ oxidation on the photoanode with concurrent hydrogen generation at the cathode.^67^ The composite CNGO/ZnSe photoanodes also exhibit better stability in both alkaline and neutral electrolyte environments (retention of 31.1 and 38.5% of the initial current density after 12 and 3 h, respectively; see Figure S17), in the absence of TEOA, compared to the bare CNGO film (27.2 and 34.1%, respectively).

A comparison table for the PEC performance using various materials supported on CN-based films is given in Table S1. It is worth mentioning that few reports show the OER FE, thus emphasizing the CNGO/ZnSe film’s superior efficiency as a photoanode. Analysis using SEM of the CNGO/ZnSe film after the 12 h stability test (Figure S18a–c) reveals minimal alterations in the thickness and morphology of the film. However, the XPS analysis discloses partial oxidation of Se (−2) into Se (+4), as shown in Figure S18d. Postoperation XRD does not show significant differences or prominent selenium oxide diffractions. However, the intensities of ZnSe relative to those of FTO diminish, possibly indicating partial oxidation of the outer layer of ZnSe into an amorphous phase (Figure S19). Additionally, C 1s and N 1s XPS spectra (Figure S20) exhibit slight shifts in their binding energy values, which is also consistent with partial oxidation of the CN framework during the stability test.

Incident photon-to-current conversion efficiency (IPCE) was measured for CNGO and CNGO/ZnSe electrodes at different illumination wavelengths in the 320–520 nm range in an alkaline environment (Figure 4f). The IPCE values exhibit a consistent correspondence with the films’ optical absorption spectra. Notably, IPCE values for the CNGO/ZnSe film are higher than bare CNGO, with a more than double increase at 375 nm, where the maximum of CNGO appears (∼9.9% vs 4.1%). Furthermore, the IPCE assessment confirms that the CNGO/ZnSe electrode’s extended absorbance in the visible range translates into photoactivity across extended wavelengths, reaching approximately 460 nm. We attribute this extension to the absorbance of ZnSe NCs and enhanced charge separation and transfer. Additionally, in the presence of TEOA, there was an increase in the IPCE value: 8.1% for the CNGO film and 16.6% for the CNGO/ZnSe film at 375 nm. The corresponding absorbed photon-to-current efficiency (APCE) values for CNGO and CNGO/ZnSe films were calculated to be 3.8 and 9.5%, respectively, at 400 nm, as illustrated in Figure S21.

## Conclusions

In summary, we have constructed a porous CNGO/ZnSe heterojunction system using the EPD method while maintaining the intimate connection of the photoactive film with the FTO substrate. The EPD of BF_4_^–^-treated ZnSe NCs on CNGO electrode yields a uniform distribution of ZnSe NCs on the CNGO surface throughout the film, which results in a stable and improved photocurrent density (160 ± 8 μA cm^–2^ at 1.23 V vs RHE in an alkaline electrolyte) in photoelectrochemical water-splitting. Spectral and photoelectrochemical measurements reveal an extended photoresponse in the visible range, lower charge recombination, the formation of charge transfer states due to the heterojunction, type-II band alignment, and charge transfer from ZnSe to CN. The CNGO/ZnSe photoanode retains close to 100% activity even after a continuous 12 h operation in 0.1 M KOH containing 10% v/v TEOA hole scavenger at 1.23 V vs RHE. The IPCE values for the CNGO/ZnSe photoanode reach ∼9.9% at 375 nm with an extended absorbance in the visible range. Moreover, the CNGO/ZnSe film exhibited 87% FE for oxygen generation during a 30 min water-splitting PEC.

The introduced strategy opens a path for forming numerous composite photoelectrodes, combining the versatility of the CN-based polymeric porous electrode design with flexibility in selecting diverse NC systems with properties controlled by size, shape, composition, and surface chemistry. The availability of all these powerful knobs shows promise for tailoring the PEC response toward specific chemical tasks with enhanced efficiencies and stability.

## Methods

### Chemicals

Zinc stearate (90%), selenourea (98%), trimethyloxonium tetrafluoroborate (95%), and melamine (99%) were purchased from Sigma-Aldrich. Oleylamine (OAm, 80–90%), dodecanethiol (98%), NaH_2_PO_4_ (96%), and Na_2_HPO_4_ (98+%) were purchased from Thermo Scientific. Dimethylformamide (DMF, ≥99.5%) was purchased from Fisher Chemical. Ethylene glycol (EG, ≥99.5%) was purchased from Merck. Ethanol (≥99.9%), toluene (99.8%), acetonitrile (99.9%), sulfuric acid (H_2_SO_4_, 98% w/w), and acetone (99.5%) were purchased from Bio-Lab Ltd., Israel. Potassium hydroxide pellets (KOH, AR grade, 85% w/w) were purchased from Loba Chemie, India. TEOA (≥99.0%) was purchased from Glentham, U.K. Fluorine-doped tin-oxide (FTO)-coated glass (12–14 Ω sq^–1^) was bought from Xop Glass Company, Spain. Graphene oxide (GO, 0.4% w/w, >95%) aqueous suspension was brought from University Wafer Inc., USA. In addition, deionized (DI) water with 18.2 MΩ cm resistivity was obtained using a Millipore Direct-Q3 water purification system. All chemicals were used as received without further purification.

### Synthesis of ZnSe NCs

The procedure is a modification of a previously reported synthesis.^57^ 940 mg of Zn stearate (1.5 mmol) and 360 mg of selenourea (3 mmol) were dissolved in 37 mL of oleylamine and 4 mL of dodecanethiol in a three necked flask. After degassing at 60 °C, the solution was heated to 250 °C under an inert environment and held at this temperature for 45 min. The reaction solution was then cooled to room temperature and cleaned from excess ligands using toluene as the solvent and a mixture of acetone and acetonitrile as antisolvents in a 5:5:1 (toluene:acetone:acetonitrile) ratio.

### ZnSe NC Ligand Stripping and Transfer to DMF

The as-synthesized ZnSe NCs were first diluted to a final concentration of ∼16 μM in toluene with a small amount of DMF (10% v/v). In parallel, a BF_4_^–^ solution was prepared by dissolving trimethyloxonium tetrafluoroborate in acetonitrile (∼1 M). The two solutions were then mixed together in a 1:1 ratio and cleaned twice, using DMF as the solvent and toluene as the antisolvent. Finally, the BF_4_^–^ surface-treated ZnSe NCs were dispersed in DMF to the desired concentration for EPD (∼4 μM, O.D. = 3 at 420 nm).

### CNGO Film Preparation and Characterization

A porous polymeric CN film with embedded reduced graphene oxide (CNGO) over fluorine-doped tin-oxide coated glass (acting as the transparent conductive substrate; referred to henceforth simply as FTO) was prepared as an electrode. Melamine and graphene oxide (GO, 0.8% w/w, obtained through the concentration via heating of 0.4% w/w GO aqueous suspension at 55 °C) mixture were dispersed using EG into a paste that was doctor-bladed (height determined by the number of used scotch tape layers, *L* = 1, 2, or 3) over the FTO, and after calcination formed the porous layer, CNGO, as depicted in Scheme 1. The CNGO film obtained from *L* = 2 is the optimized film thickness (quality of contact to the FTO and light absorption), yielding the best PEC activity, thus making it the preferred choice for this study.^28^ In our previous study, we investigated the role of rGO, demonstrating that its presence notably enhances charge transfer, increases the electrochemical active surface area, extends electron diffusion length, and consequently improves the overall activity.^17^

### ZnSe NC Deposition into the Porous CNGO Film via EPD

EPD of the ligand-stripped ZnSe NCs onto the porous CNGO film was performed in DMF.^68^ A parallel capacitor configuration (distance ∼ 3 mm) between FTO and FTO/CNGO substrates was used, and 80 V was applied for 5, 10, or 15 s in a glass vessel with a total volume of 3 mL (4 μM) of ZnSe NC solution. During the deposition process, we optimized parameters such as voltage (50, 80, and 100 V) and initial concentration (2, 4, and 8 μM) of the NC solution. Deposition of NCs occurred on the CNGO-coated FTO negatively biased electrode. A drying procedure was developed to prevent cracks: the electrodes were heated to 35 °C in a vacuum oven overnight, before further characterization or use as FTO/CNGO/ZnSe photoanodes.

## Supporting Information Available

Detailed characterization procedures, ZnSe NC structural and optical characterization, CNGO and CNSO/ZnSe photoelectrode compositional, optical, and (photo)electrochemical characterizations, transient absorption measurements, chronoamperometric measurements in different electrolytes (pH, hole scavenger presence), stability test and post-PEC characterization, and performance comparison of nanoparticle-loaded CN photoanodes for water-splitting (PDF)
