# Supplementary material for: NC Meets CN: Porous Photoanodes with Polymeric Carbon Nitride/ZnSe Nanocrystal Heterojunctions for Photoelectrochemical Applications
Source: ACS Appl Mater Interfaces. 2024 Jul 16;16(29):38153–62. doi: 10.1021/acsami.4c07582 (PMC11284784; doi:10.1021/acsami.4c07582)
Supplement: Supplementary file 1 — am4c07582_si_001.pdf [file am4c07582_si_001.pdf]

Supporting Information

NC Meets CN: Porous Photoanodes with Polymeric Carbon Nitride/ZnSe Nanocrystal Heterojunctions for Photoelectrochemical Applications

Sanjit Mondal[†][a], *Tom Naor*[‡][a], *Michael Volokh*[†], *David Stone*[‡], *Josep Albero*[₴], *Adar Levi*[‡], *Atzmon Vakahi*[‡], *Hermenegildo García*[₴], *Uri Banin*[‡]*, *Menny Shalom*[†]*

† Department of Chemistry and Ilse Katz Institute for Nanoscale Science and Technology, Ben-Gurion University of the Negev, Beer-Sheva 8410501, Israel.

E-mail: mennysh@bgu.ac.il

‡ The Institute of Chemistry and The Center for Nanoscience and Nanotechnology, The Hebrew University of Jerusalem, Jerusalem 91904, Israel.

E-mail: uri.banin@mail.huji.ac.il

₴ Instituto Universitario de Tecnología Química CSIC-UPV, Universitat Politècnica de València, València 46022, Spain.

E-mail: hgarcia@qim.upv.es

[a] Sanjit Mondal and Tom Naor have contributed equally to this work

Supporting Information Table of Content

Figure S1: Characterization of ZnSe NCs. (a) TEM and (b) HRTEM imaging of post-synthesis ZnSe NCs. (c) TGA, (d) UV–vis absorbance spectra, and (e) XRD patterns of ZnSe NCs, before (OAm, red) and after (BF_4, yellow) surface treatment. (**Page S9**)

Note S1: Surface site calculation. (**Page S9**)

Figure S2: (a) XRD patterns of the electrodes. (b) High-resolution Zn 2p and Se 3d XPS spectra of CNGO/ZnSe film. (**Page S10**)

Figure S3: (a, b) C 1s and N 1s XPS of film CNGO film respectively. (c, d) C 1s and N 1s XPS of film CNGO/ZnSe film respectively. (**Page S10**)

Figure S4: FTIR spectra of CNGO and CNGO/ZnSe films (different EPD duration). (**Page S11**)

Figure S5: UV–vis DRS plots of films over FTO: CNGO, CNGO/ZnSe (different EPD duration—5 s, 10 s, and 15 s), and ZnSe nanocrystals only. (**Page S11**)

Note S2: Valence band XPS calculation. (**Page S12**)

Figure S6: Mott–Schottky plots of CNGO and CNGO/ZnSe films. (**Page S12**)

Figure S7. Nyquist plots from EIS measurements of CNGO and CNGO/ZnSe films. (a, b) fitted and raw data of CNGO film respectively. (c, d) fitted and raw data of CNGO/ZnSe films respectively. (e) Plot of charge transfer resistance (R_{ct}) values obtained for CNGO and CNGO/ZnSe films at different potential. (f) Equivalent circuit used for the fitting. (**Page S13**)

Figure S8: Open circuit potential (V_{OC}) for CNGO and CNGO/ZnSe films under dark and 1 sun illumination. (**Page S14**)

Figure S9. (a) Transient absorption spectra of CNGO, ZnSe, and CNGO/ZnSe dispersions in N_2-saturated acetonitrile at the delay time of 250 ns. (b, c) Transient absorption decay of CNGO, ZnSe, and CNGO/ZnSe, monitored at (b) 375 nm and (c) 500 nm, respectively. (**Page S15**)

Note S3: Transient absorption (TA) spectroscopic studies. (**Page S15–S16**)

Figure S10. Cyclic voltammetry test of the CN, CNGO and CNGO/ZnSe films. (**Page S17**)

Figure S11: Applied bias photon-to-current conversion efficiency (ABPE) of CNGO and CNGO/ZnSe as a function of applied potential. (**Page S17**)

Figure S12: Chronoamperometric measurements of CNGO/ZnSe film in electrolyte solutions of different pH values. All measurements at 1.23 V *vs.* RHE with cyclic on/off 1 sun illumination. (**Page S18**)

Figure S13: (a) FE plot for the O_2 production. (b) The chronoamperometric measurement of CNGO and CNGO/ZnSe films, recorded during the gas production measurement. (**Page S18**)

Figure S14: (a) Measured H_2 production for CNGO and CNGO/ZnSe films. (b) The corresponding FE plot for the H_2 production. (**Page S19**)

Figure S15: (a, b) Three sets of chronoamperometry measurement data obtained in 0.1 M KOH at 1.23 V *vs.* RHE for CNGO and CNGO/ZnSe films respectively. (c, d) Three sets of chronoamperometry measurement data obtained in 0.1 M KOH solution containing 10% v/v TEOA hole scavenger at 1.23V *vs.* RHE for CNGO and CNGO/ZnSe films respectively. (**Page S19**)

Figure S16: Chronoamperometric measurements of CNGO and CNGO/ZnSe films with and without hole scavenger in 0.1 M aqueous KOH electrolyte at 1.23 V *vs.* RHE with cyclic on/off 1 sun illumination. (**Page S20**)

Figure S17: Chronoamperometric stability of CNGO and CNGO/ZnSe (10 s) films. (a) in alkaline 0.1 M KOH and (b) in a neutral phosphate buffer. All measurements at 1.23 V *vs*. RHE under constant 1 sun illumination. (**Page S20**)

Table S1: Comparison of nanoparticle-loaded polymeric carbon nitride photoanodes used for water-splitting. (**Page S21**)

Figure S18: Characterization of a CNGO/ZnSe film after the stability test. (a, b) Top–view and (c) cross-sectional SEM images. (d) High-resolution Se 3d XPS spectra before and after the stability test. (**Page S22**)

Figure S19: PXRD pattern of CNGO/ZnSe film after the stability experiment. (**Page S22**)

Figure S20: Characterization of a CNGO/ZnSe film after the stability test. (a) C 1s, and (b) N 1s XPS spectra. (**Page S23**)

Figure S21: Absorbed photon-to-current efficiency (APCE) of CNGO and CNGO/ZnSe films. (**Page S24**)

Characterization: The morphology analysis of the synthesized ZnSe nanorods (NRs) was conducted using transmission electron microscopy (TEM) on a Tecnai G2 Spirit Twin T12 microscope (Thermo Fisher Scientific) operated at an accelerating voltage of U_0 = 120 kV. Additionally, high-resolution TEM (HRTEM) measurements were performed using a Tecnai F20 G2 microscope (Thermo Fisher Scientific) at U_0 = 200 kV. The extinction coefficient of ZnSe NRs was calculated using a previously reported method.[1] The structural analysis of synthesized photoelectrodes was performed using powder X-ray diffraction patterns (XRD) recorded by a PANalytical's Empyrean diffractometer, equipped with a position-sensitive detector X'Celerator. Data was recorded with a scanning time of ~15 min for 2θ ranging from 10° to 60° using Cu Kα radiation (λ = 1.54178 Å, 40 kV, 30 mA). Fourier-transform infrared spectroscopy (FTIR) was carried out to study the functional groups of the electrode materials on a Thermo Scientific Nicolet iS5 FTIR spectrometer (equipped with a Si attenuated total reflectance (ATR) accessory). X-ray photoelectron spectroscopy (XPS) measurements were performed on a Thermo Scientific ESCALAB 250 (Al Kα, 1486.6 eV) with an applied pass energy 20 eV. UV–vis absorption and steady-state photoluminescence (PL) spectroscopies were used to study the optical properties of the electrode materials. A Cary 100 spectrophotometer, in a double-beam configuration using two 10 mm quartz cuvettes for liquids or equipped with a diffuse reflectance accessory (DRA) for powder and films was used for UV–vis absorption studies. A Horiba Scientific FluroMax 4 spectrofluorometer was used for steady-state PL spectroscopy. The valence band (VB) maximum energy was estimated using XPS measurements in a Thermo Scientific ESCALAB Xi+ with a HeI excitation source. The morphology of the supramolecular precursor and the final photoelectrodes were characterized by scanning

electron microscopy (SEM) using a FEI Verios ultrahigh-resolution SEM (equipped with a FEG source and a TLD detector), operated at U_0 = 3.5 kV and I = 25 pA; to avoid charging effects, some samples were sputtered with ≤ 5 nm Au-Pd alloy using a Quorum Q150T ES system. Depth profile imaging was performed using an SEM instrument equipped with a dual-beam focused ion beam (FIB – Helios Nanolab 460F1Lite Dual Beam FIB/SEM, Thermo Fisher), using Ga ions, accelerated at 30 kV with 230 pA current, while the SEM microscope was operated at U_0 = 2.0 kV and I = 0.1 nA. Transient absorption spectroscopy experiments were carried out in N_2-saturated dispersions of the samples in acetonitrile. The experiments were carried out using an OPO system Ekspla (EKS-NT342C-10) coupled with a UV extension (EKS-NT342C-SH-SFG) as the excitation source and an Edinburgh Instruments detection system (LP980) coupled with an ICCD camera (Andor iStar CCD 320 T).

PEC and electrochemical measurements: All the photoelectrochemical measurements were carried out using a standard three-electrode system on a single-channel PalmSens4 potentiostat (PalmSens, Netherlands). A Pt foil (1.0 cm^2) and Ag/AgCl (saturated KCl) were used as the counter- and reference-electrodes, respectively. 0.10 M KOH aqueous solution, (pH ~13.1) or 0.1 M KOH aqueous solution containing 10% v/v triethanolamine were used as the electrolyte for the photocurrent experiments. Additionally, phosphate buffer and 0.50 M H_2SO_4 aqueous solutions were also used for photocurrent measurements in neutral and acidic environments, respectively. The measured potentials ($V_{Ag/AgCl}$) were converted to the reversible hydrogen electrode (RHE) scale using the following equation:

$$V_{RHE} = V_{Ag/AgCl} + 0.0591 \times pH + 0.197\ V \qquad \text{(Eq. S1)}$$

Photocurrent density of the films was measured at a bias potential of V_{RHE} = 1.23 V under 1 sun illumination (power density of ~100 mW cm^{-2}) supplied by a Newport LCS-100 solar simulator (100 W Xe lamp and an integrated AM 1.5 filter, calibrated using a Newport 919P power meter). All PEC measurements were taken at consistent intervals of 20 seconds, alternating between light on and light off conditions. Linear sweep voltammetry (LSV) measurements were performed in the dark and under 1 sun illumination in the range of 0–1.8 V *vs.* RHE. Applied-bias photon-to-current conversion efficiency (ABPE) was calculated using the following equation:

$$\text{ABPE (\%)} = \frac{J \times (1.23 - |V_b|)}{P_{\text{incident}}} \times 100\% \qquad \text{(Eq. S2)}$$

Where J is the current density (measured in mA cm^{-2}) obtained under an applied bias V_b (*vs.* RHE), measured in V. $P_{incident}$ is the total incident illumination power density (source: the solar simulator) measured in mW cm^{-2}.

For incident photon-to-current conversion efficiency (IPCE) measurements, a Zahner CIMPS-QE/IPCE photoelectrochemical workstation coupled with a TLS03 tunable light source controlled by a PP211 potentiostat (Zahner-Elektrik, Germany) in a dedicated three-electrode photoelectrochemical cell (PEEC-2) using an Ag/AgCl (sat. KCl) reference electrode and Pt coil as the counter electrode was used. The IPCE calculations were performed using the following equation:

$$\text{IPCE (\%)} = \frac{J \times 1240}{\lambda \times I_{\text{incident}}} \times 100\% \qquad \text{(Eq. S3)}$$

Where J is the photocurrent density in units of mA cm^{-2}, 1240 is the units conversion factor, $I_{incident}$ is the incident illumination power in units of mW cm^{-2} (calibrated to

illumination spot of 8 mm in diameter) of the specific monochromatic LED illumination wavelength (λ is measured in nm). The calculation was performed by the coupled ThalesXT software.

Absorbed photon-to-current efficiency (APCE) estimation for each wavelength was calculated from the ratio between the wavelength-dependent IPCE to the fraction of absorbed photons at that wavelength:

$$\text{APCE}\ (\%) = \frac{\text{IPCE}\ (\%)}{100\% - R - T} \times 100\% \qquad \text{(Eq. S4)}$$

Where R (reflection) and T (transmission) represent the %reflection and %transmission losses out of the total incident illumination, *i.e.*, the %absorbance is (A = 100% – T – R).

Mott–Schottky analysis was performed in 0.5 M Na_2SO_4 aqueous solution using an Autolab potentiostat (PGSTAT302N, Metrohm, Switzerland). The carrier concentration (N_D) was calculated from a Mott–Schottky analysis at a 1 kHz frequency using the following equation:

$$N_\text{D} = \frac{2}{ne\varepsilon\varepsilon_0} \qquad \text{(Eq. S5)}$$

Where n is the slope of the linear part of the C^{-2} *vs.* V_{RHE} plot (Figure S6), $e = 1.602 \times 10^{-19}$ C (electron charge), $\varepsilon_0 = 8.860 \times 10^{-12}$ F m^{-1} (vacuum permittivity), and $\varepsilon = 9.801$ (relative permittivity) for carbon nitride.[2]

EIS measurements (three-electrode configuration) in 0.5 M Na_2SO_4 aqueous solution were used to measure the complex impedance of the photoanodes in the dark (films over FTO). The Nyquist plots represent measurements at applied potentials of 0.05,

0.1, 0.15, and 0.25 V *vs.* RHE over a frequency range from 50 kHz to 100 mHz using PalmSens4 potentiostat (PalmSens, Netherlands).

Evolved gas quantification during water-splitting (OER and HER) experiments: Oxygen (O_2) generation for CNGO and CNGO/ZnSe NCs films in 0.1 M KOH solution was detected using a fiber optic oxygen meter (Firesting GmbH, Germany) under chronoamperometric condition (1.23 V *vs.* RHE, 1 sun illumination) in an H-cell configuration. The two-compartment cell (H-cell) was tightly sealed with a rubber septum and parafilm to avoid gas leakage. The electrolyte solution was purged with Ar (99.999%) for 30 min before the experiments. The O_2 quantification was performed for a duration of 1 hour, as shown in Figure 4c in the manuscript. The presented data was acquired after background subtraction. For H_2 quantification, 100 µL sample of gas was taken every 20 min with an A-2 Luer lock gas syringe (Pressure-lok precision analytical syringe from VICI), sampling from the headspace and injected into a gas chromatograph (Agilet 7820 GC system), equipped with a CP-Molsieve 5A column. The Faradaic efficiency (FE) was calculated using Eq. S6–S7:

$$n = \frac{I \times t}{z \times F} \qquad \text{(Eq. S6)}$$

Where n is the gas amount (measured in mol), I stands for the current (A), z is the number of transferred electrons (for O_2 (OER) and H_2 (HER), z = 4 and 2, respectively), t is the time (s), and F is the Faraday constant (96,485C mol^{-1}).

The theoretical amount of O_2 and H_2 was calculated from Faraday's law, Eq. S7:

$$\text{FE (\%)} = \frac{\text{Experimental evolved gas amount}}{\text{Theoretical evolved gas amount}} \times 100\% \qquad \text{(Eq. S7)}$$

Where the evolved gasses (O_2 or H_2) were quantified in µmol.

Supporting Information Figures, Notes, and Tables

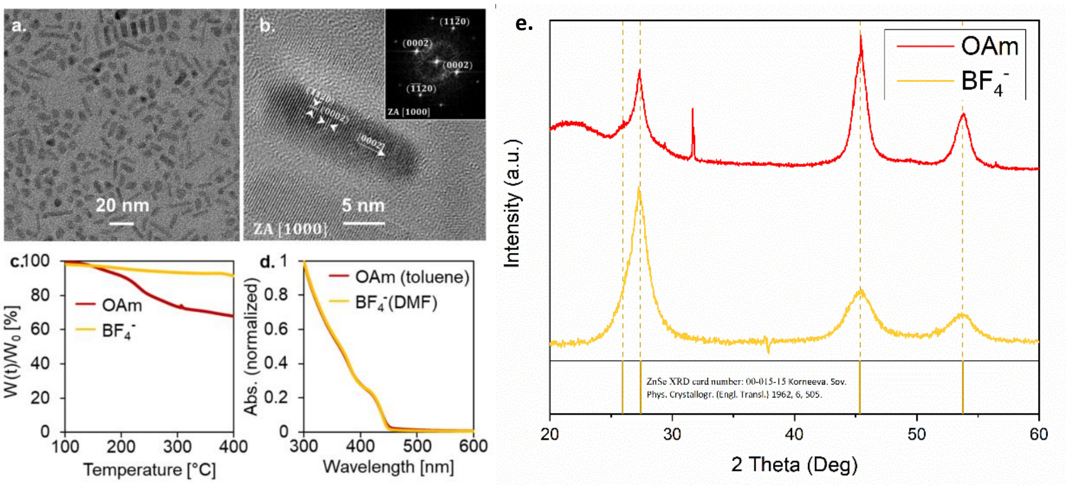

Figure S1. Characterization of ZnSe NCs. (a) TEM and (b) HRTEM imaging of ZnSe NCs. (c) TGA, (d) UV–vis spectra, and (e) XRD patterns of ZnSe NCs, before (capped with OAm, red trace) and after (BF_4^- on the surface, yellow trace) surface treatment. The UV–vis absorption spectra are normalized to the absorbance value at a wavelength of 300 nm.

Note S1. Surface site calculation.

The number of Zn surface sites in a nanorod ($N_{surface}$) was calculated based on a previously reported method,[2] assuming simple cylinder model for the NCs (cylinder radius and height were derived from TEM statistical analysis). These NCs are organized in a wurtzite structure with lattice parameters of c = 6.506 Å and u = 0.375.[3]

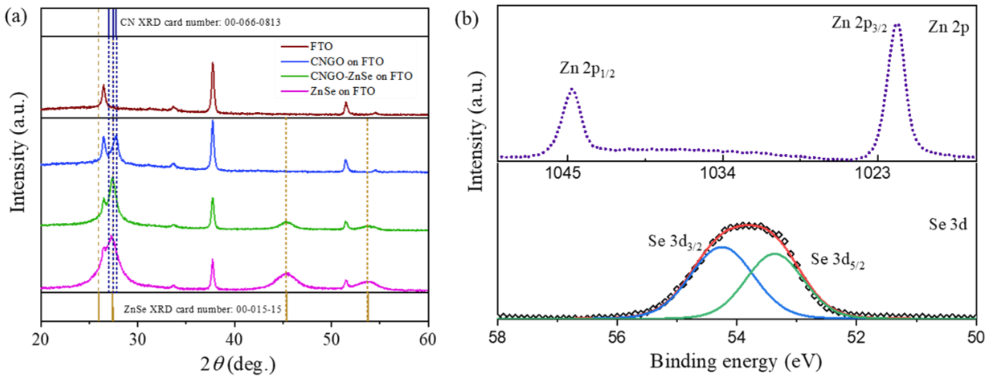

Figure S2. (a) XRD patterns of the electrodes. The hexagonal ZnSe reference stick pattern (wurtzite ZnSe) is based on XRD card number 00-015-0105. For CN reference pattern is based on XRD card number 00-066-0813. (b) High-resolution Zn 2p and Se 3d XPS spectra of CNGO/ZnSe film.

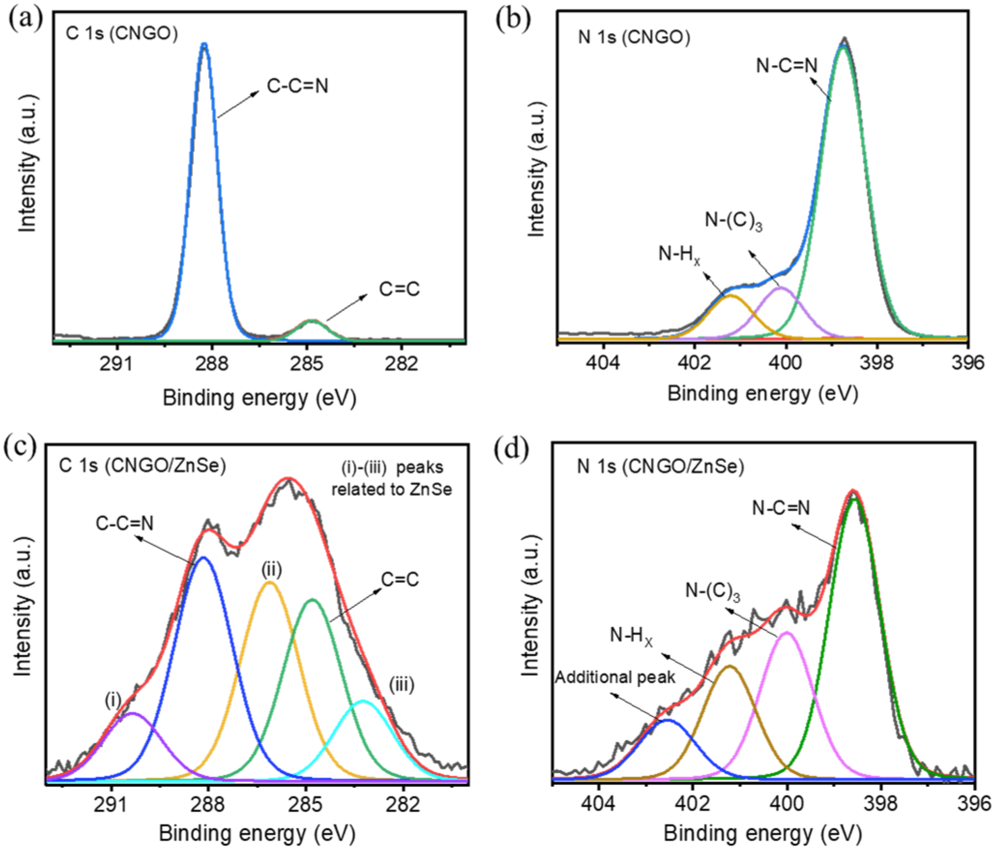

Figure S3. XPS analysis of films. (a) C 1s and (b) N 1s of CNGO. (c) C 1s and (d) N 1s XPS of CNGO/ZnSe.

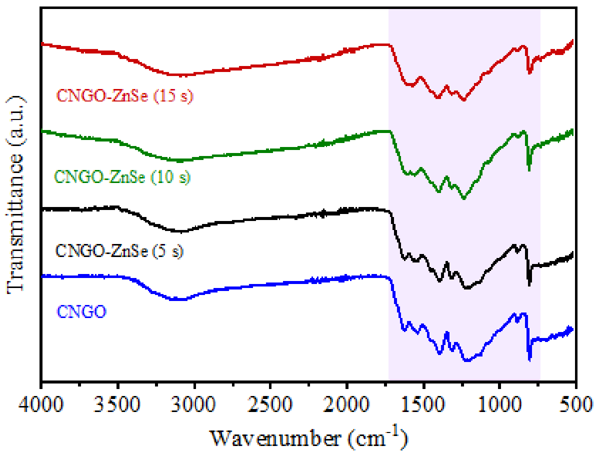

Figure S4. FTIR spectra of CNGO and CNGO/ZnSe films (different EPD duration).

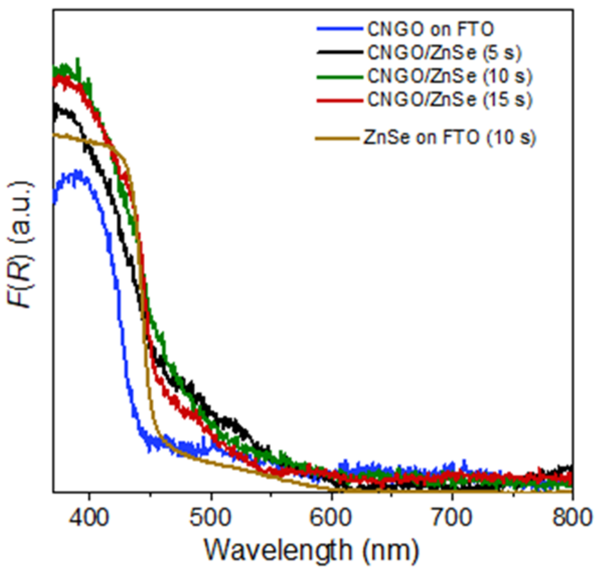

Figure S5. UV–vis DRS plots of films over FTO: CNGO, CNGO/ZnSe (different EPD duration—5 s (black), 10 s (green), and 15 s (red)), and ZnSe nanocrystals only (brown).

Note S2. XPS valence band values conversion to the NHE scale.

To convert to the measured XPS VB values to the normal hydrogen electrode (NHE) scale, the following calculation was performed for the electrodes using Eq. S8:

$$E_{\mathrm{NHE}}\ (\mathrm{V}) = \Phi + E_{\mathrm{VB\text{-}XPS}} - 4.44 \qquad \text{(Eq. S8)}$$

Where Φ is the work function of the instrument (Φ = 4.84 eV), $E_{\mathrm{VB\text{-}XPS}}$ is the measured valence band maximum energy value, and 4.44 eV is the vacuum level.

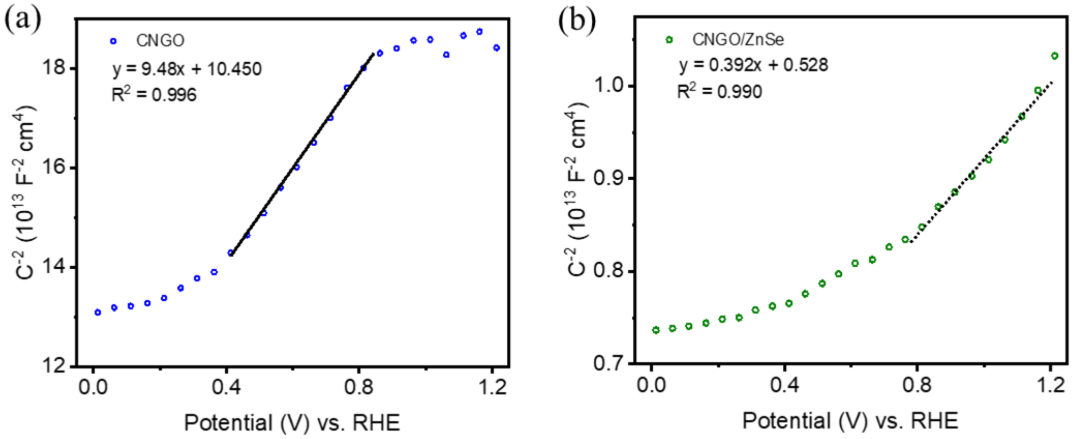

Figure S6. Mott–Schottky plots of (a) CNGO and (b) CNGO/ZnSe films. The calculated slopes from the linear regression are: 9.48×10^{13} and 3.92×10^{12} $cm^4\ F^{-2}\ V^{-1}$ for CNGO, and CNGO/ZnSe, respectively.

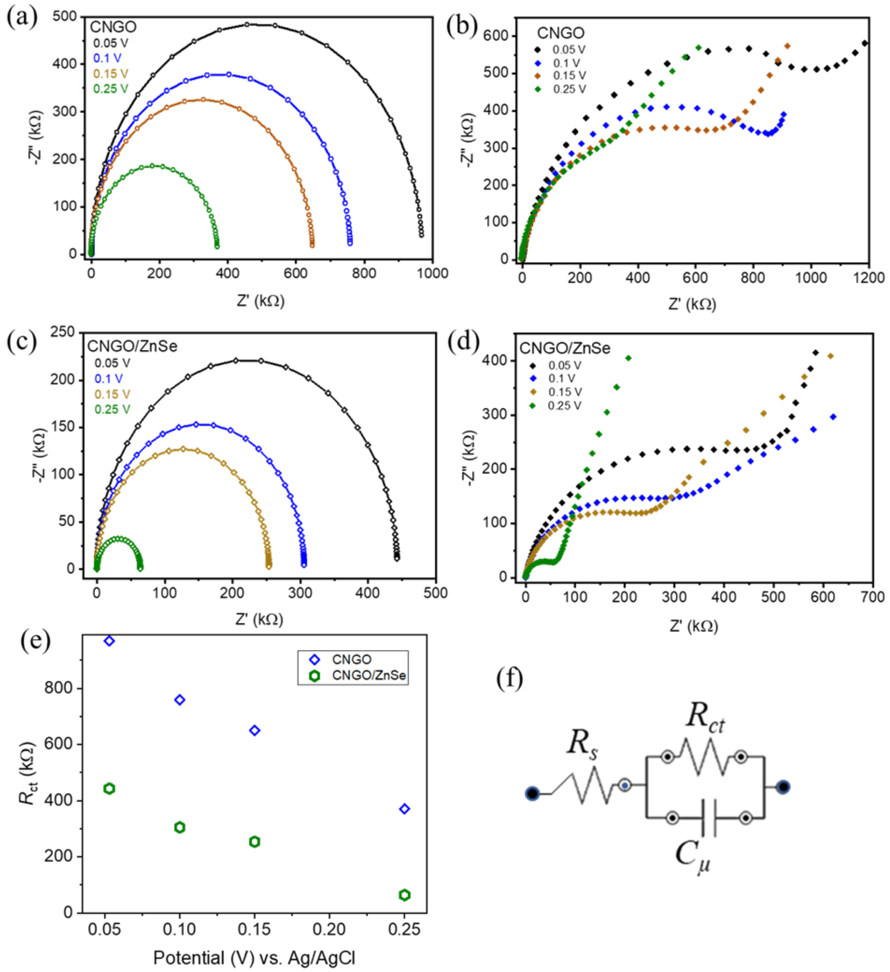

Figure S7. Nyquist plots from EIS measurements of CNGO and CNGO/ZnSe films. (a) fitted and (b) experimental data points of CNGO. (c) fitted and (d) experimental data points of CNGO/ZnSe. (e) Plot of calculated charge transfer resistance (R_{ct}) values obtained for CNGO and CNGO/ZnSe films at different potential from the fitted data. (f) The equivalent circuit used for fitting.

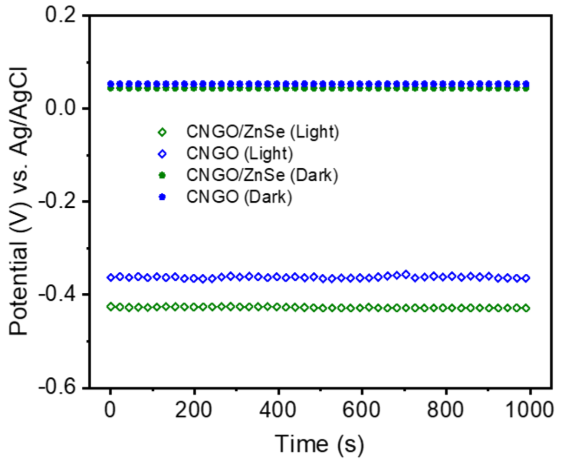

Figure S8. Chronopotentiometric measurements showing the open circuit potential (V_{OC}) for CNGO and CNGO/ZnSe films under dark (circles) and under 1 sun illumination (empty rhombi). Measurements were carried out using Ag/AgCl (saturated KCl) reference electrode in 0.1 M aqueous KOH electrolyte solution purged with Ar for 20 minutes before the measurement.

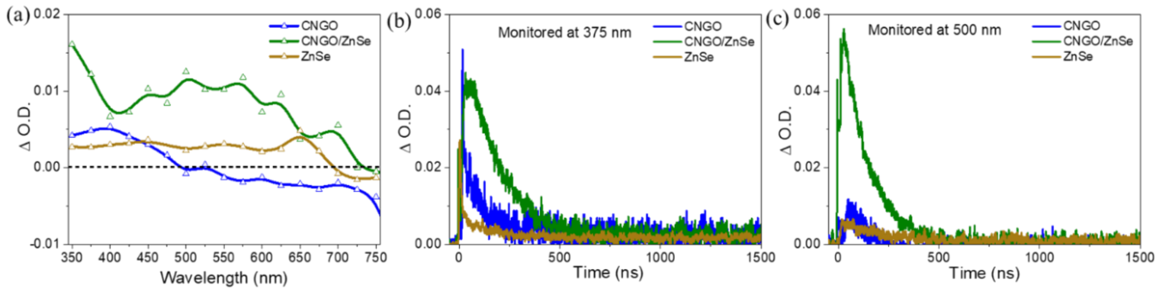

Figure S9. (a) Transient absorption spectra of CNGO (blue), ZnSe (yellow), and CNGO/ZnSe (green) dispersions in N_2-saturated acetonitrile at the delay time of 250 ns. (b, c) Transient absorption decay of CNGO (blue), ZnSe (brown), and CNGO/ZnSe (green), monitored at (b) 375 nm and (c) 500 nm, respectively. Laser excitation wavelength is 300 nm. The absorbance of all samples was identical at 300 nm.

Note S3. Transient absorption (TA) spectroscopic studies.

The TA spectra of CNGO/ZnSe, pristine CNGO, and ZnSe dispersions in N_2-saturated acetonitrile were measured upon 300 nm laser excitation, acquired at 250 ns. As shown in Figure S9a, CNGO exhibits a positive TA band spanning from 350 nm up to 500 nm, with a maximum centered at 400 nm. In contrast, ZnSe displays a continuous TA signal extending from 350 nm to 700 nm. Interestingly, CNGO/ZnSe presents a distinct TA spectrum, featuring a series of bands. The first and most intense—extends from the UV region up to 400 nm—has a maximum at wavelengths shorter than 350 nm, while there are five TA bands throughout the entire visible spectrum, the most intense one appearing *ca.* 525 nm. Furthermore, the increased optical density (O.D.) observed in the CNGO/ZnSe TA spectrum, recorded at the same laser power and O.D. of the suspension at 300 nm, relative to CNGO and ZnSe samples suggests a higher concentration of excited states, indicative of enhanced charge separation efficiency within the heterojunction. This finding aligns well with the information obtained from the photocurrent and photoluminescence measurements presented in Figure 4 (in the manuscript) showing higher current extraction and lesser electron/hole recombination when a CNGO/ZnSe heterojunction is formed.

The TA decays monitored at 375 nm in the UV and at 500 nm in the visible region are shown in Figure S6 b and c, respectively. At 375 nm, the CNGO transient signal decay indicates two different kinetics, necessitating fitting to two consecutive first-order decays

(Eq. S9). The fitting of experimental data to Eq. S9, corresponds to a fast decay with a lifetime of τ_1 = 86 ns and a slower component with lifetime of τ_2 = 326 ns. In contrast, the ZnSe TA decay displays a single first order kinetics, fitting well to a single exponential function (Eq. S10), with a lifetime of τ_3 = 122 ns.

Remarkably, the CNGO/ZnSe transient signal decay also exhibits two distinct kinetics, as evidenced by the adequate fitting to Eq. S6, resulting in lifetimes of τ_1 = 206 ns and τ_2 = 627 ns for the fast and slow decay, respectively. Interestingly, these lifetimes measured for CNGO/ZnSe are approximately twice longer than those determined for CNGO, indicating slower recombination kinetics.

$$F(t) = A_1 e^{-\tau_1 t} + A_2 e^{-\tau_2 t} \qquad \text{(Eq. S9)}$$

$$F(t) = A_3 e^{-\tau_3 t} \qquad \text{(Eq. S10)}$$

Where, τ_i represent the emission lifetimes and A_i represent the amplitudes of different emission lifetimes.

In contrast, at 500 nm, the three samples exhibited transient signal decays fitting well with a single first-order kinetics, with lifetimes of τ_{CNGO} = 102 ns, $\tau_{CNGO/ZnSe}$ = 179 ns, and τ_{ZnSe} = 127 ns for CNGO, CNGO/ZnSe and ZnSe, respectively. The lack of coincidence in the signals' temporal profiles measured at 350 and 500 nm indicates that there must be at least two different transient species, decaying differently. This agrees with the photogeneration of electrons and holes having distinct absorption spectra.

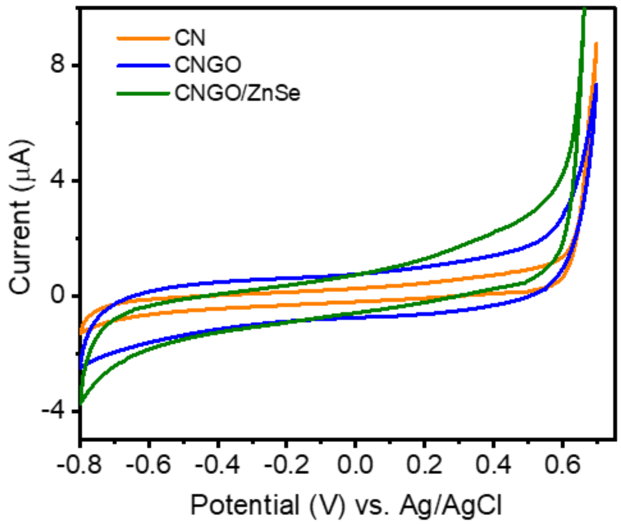

Figure S10. Cyclic voltammetry of CN, CNGO, and CNGO/ZnSe films in the dark. The measurements were carried out from –0.8 to 0.7 V *vs.* Ag/AgCl (saturated KCl) in 0.1 M aqueous KOH electrolyte at a scan rate of 50 mV s^{-1}.

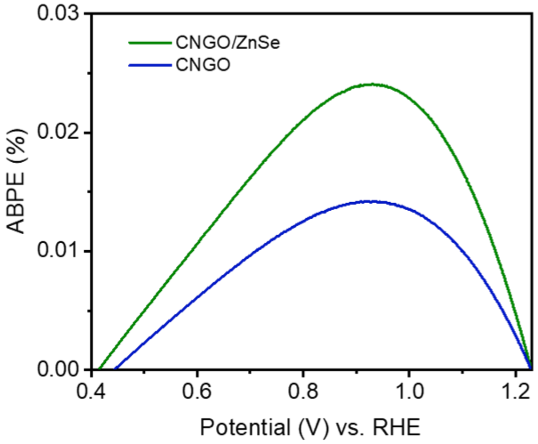

Figure S11. Applied bias photon-to-current conversion efficiency (ABPE) of CNGO and CNGO/ZnSe photoanodes as a function of applied potential.

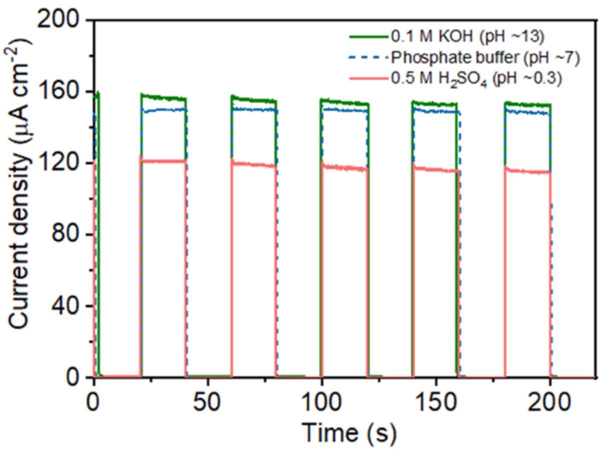

Figure S12. Chronoamperometric measurements of CNGO/ZnSe film in electrolyte solutions of different pH values. All measurements at 1.23 V *vs.* RHE with cyclic on/off 1 sun illumination.

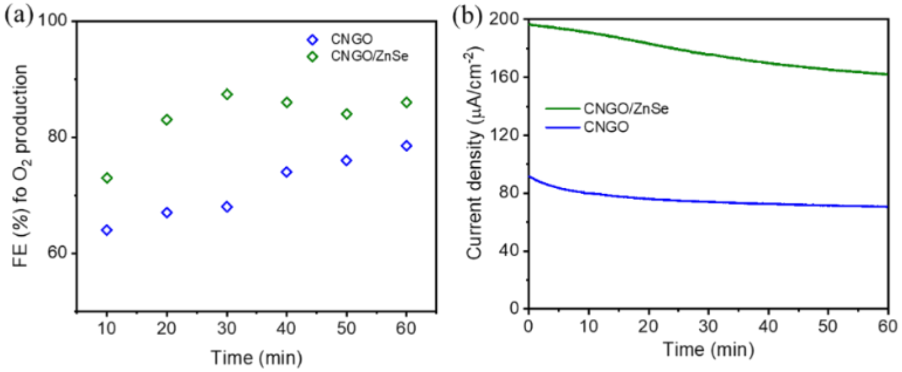

Figure S13. (a) FE plot of O_2 production. (b) The chronoamperometric measurement of CNGO and CNGO/ZnSe films, recorded during the gas production measurement.

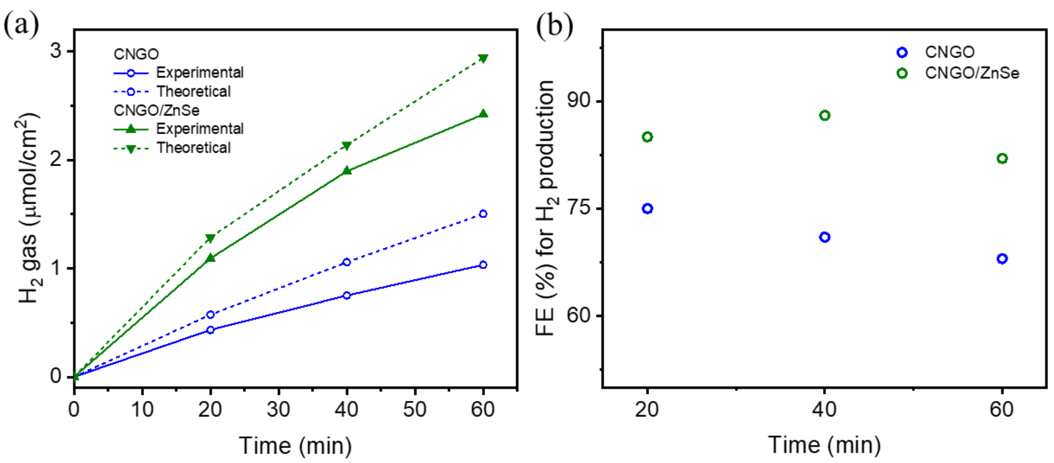

Figure S14. (a) Measured H_2 production for CNGO and CNGO/ZnSe films (*i.e.*, experimental values) and the theoretical expected values from chronoamperometry. (b) The corresponding FE plot for the H_2 production.

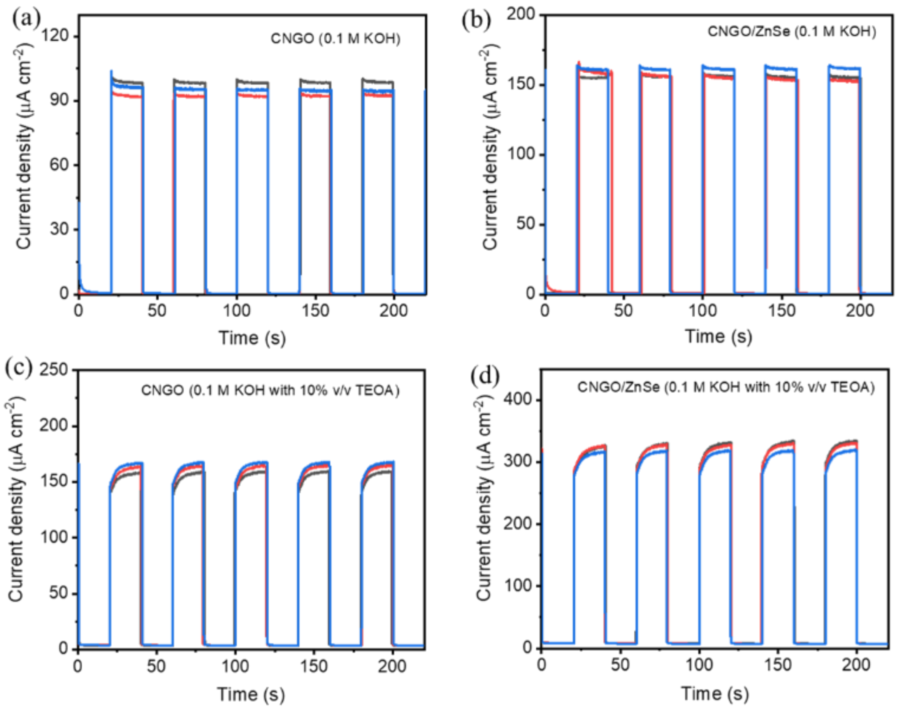

Figure S15. Chronoamperometric measurements of CNGO and CNGO/ZnSe films over FTO as photoanodes. (a) CNGO and (b) CNGO/ZnSe in three separate chronoamperometric measurements (different photoanodes, different reaction solutions) obtained in 0.1 M KOH at 1.23 V *vs.* RHE. Three sets of chronoamperometry measurements in the presence of a hole scavenger: (c) CNGO

and (d) CNGO/ZnSe in 0.1 M KOH solution containing 10% v/v TEOA (serving as the hole scavenger) at 1.23V *vs.* RHE.

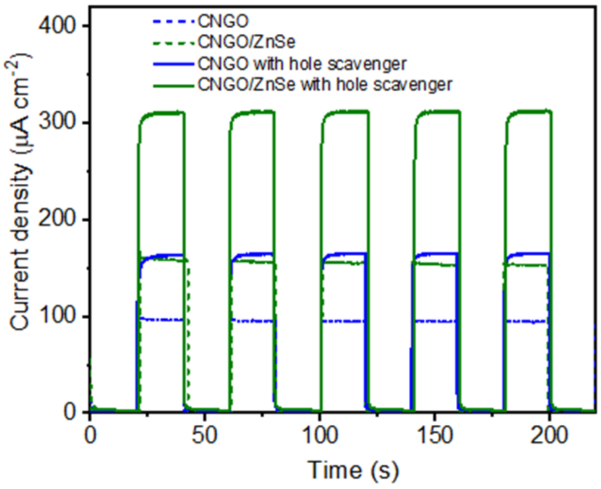

Figure S16. Chronoamperometric measurements of CNGO and CNGO/ZnSe films with and without hole scavenger in 0.1 M aqueous KOH electrolyte. All measurements at 1.23 V *vs.* RHE with cyclic on/off 1 sun illumination.

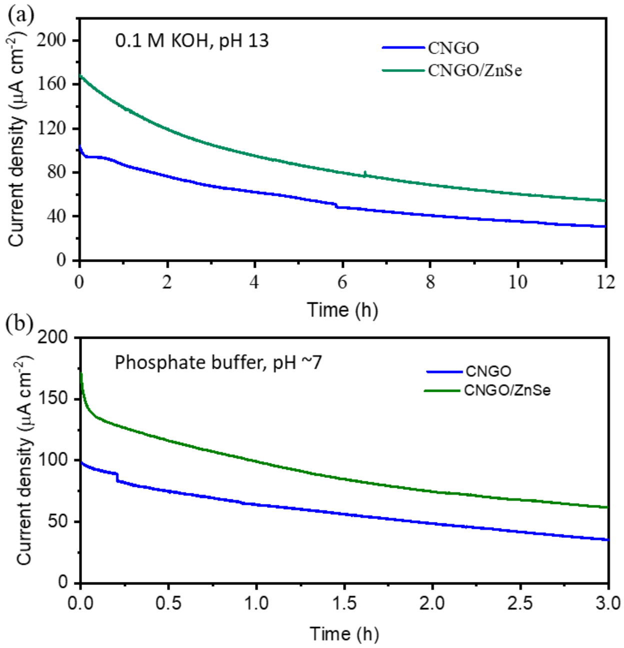

Figure S17. Chronoamperometric stability of CNGO and CNGO/ZnSe (10 s) films as photoanodes in a water-splitting PEC. (a) In alkaline 0.1 M KOH (pH ~ 13) and (b) in a neutral (pH ~ 7) phosphate buffer. All measurements at 1.23 V *vs*. RHE under constant 1 sun illumination.

Table S1. Comparison of nanocrystal-loaded polymeric carbon nitride photoanodes used for water-splitting.

Entry	Material	Electrolyte	Photocurrent (μA cm^{-2}) at 1.23 V *vs.* RHE	Faradaic efficiency (%)	Stability	Light intensity	Ref.
1	CNGO/ZnSe NCs	0.1 M KOH (pH~13)	160 ± 8	87	~100% activity after 12 h (with TEOA)<br>~40% activity after 12 h (without TEOA)	100 mW cm^{-2} AM 1.5	This work
		Phosphate buffer (pH~7)	155± 8		~37% activity after 12 h (without TEOA)		
2	g-C_3N_4/SnO_2	0.5 M Na_2SO_4	~150	—	~60% activity after 1 h	100 mW cm^{-2} AM 1.5 G	5
3	CuO/CN	0.1 M NaOH	172	40.3	—	100 mW cm^{-2} AM 1.5 G	6
4	MoS_2/g-C_3N_4	0.1 M PBS	0.4	—	—	150 W Xe AM 1.5 G	7
5	Ni-CN_x	0.1 M KOH	69.8	—	0.33 h	100 mW cm^{-2} AM 1.5	8
6	3DB WO_3-NA/C_3N_4-NS//CoO_x	0.01 M Na_2SO_4	1.5×10^3	82.8	—	150 mW cm^{-2} AM 1.5 G	9
7	Co/S-gC_3N_4/BiOCl	0.5 M Na_2SO_3 + $NaHCO_3$	393	—	3 h	150 mW cm^{-2} AM 1.5 G	10
8	Pd@g-C_3N_4	0.5 M Na_2SO_4	79	—	0.3 h	Xe lamp	11
9	g-C_3N_4-$CoFe_2O_4$	0.1 M KOH	~5	—	1 h	150 mW cm^{-2} AM 1.5 G	12
10	CoP/g-C_3N_4	0.5 M Na_2SO_4	150	—	—	100 mW cm^{-2} AM 1.5 G	13
11	ZnSe/CN	0.5 M Na_2SO_4	~1	—	0.27 h	100 mW cm^{-2} AM 1.5 G	14
12	Ag NPs loaded CN	0.5 M Na_2SO_4	~1.2	—	—	90 mW cm^{-2} AM 1.5 G	15
13	g-CN-SiC	0.5 M Na_2SO_4	~0.7	—	0.54 h	100 mW cm^{-2} AM 1.5 G	16

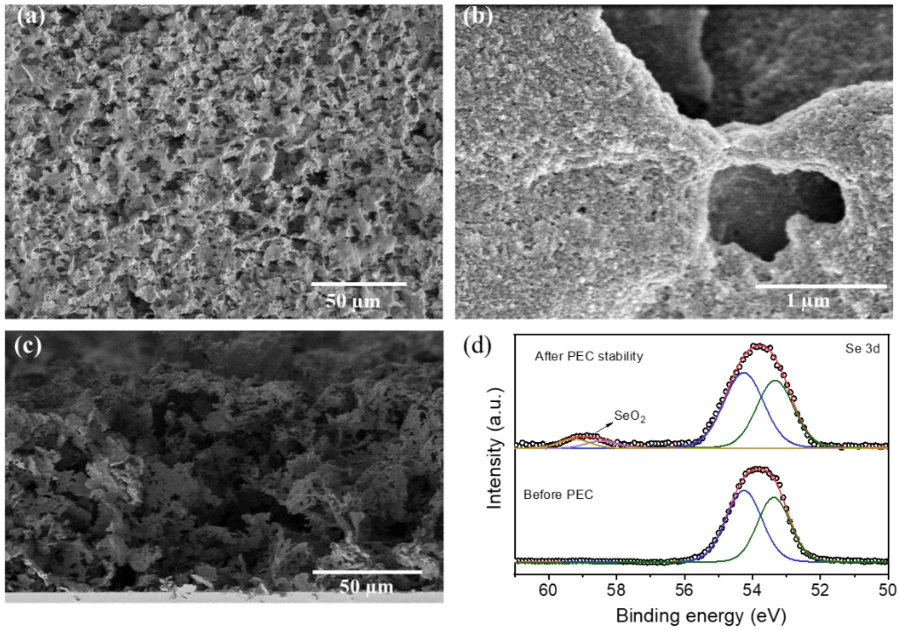

Figure S18. Characterization of a CNGO/ZnSe film after the stability test. (a, b) Top–view and (c) cross-sectional SEM images. (d) High-resolution Se 3d XPS spectra before and after the stability test.

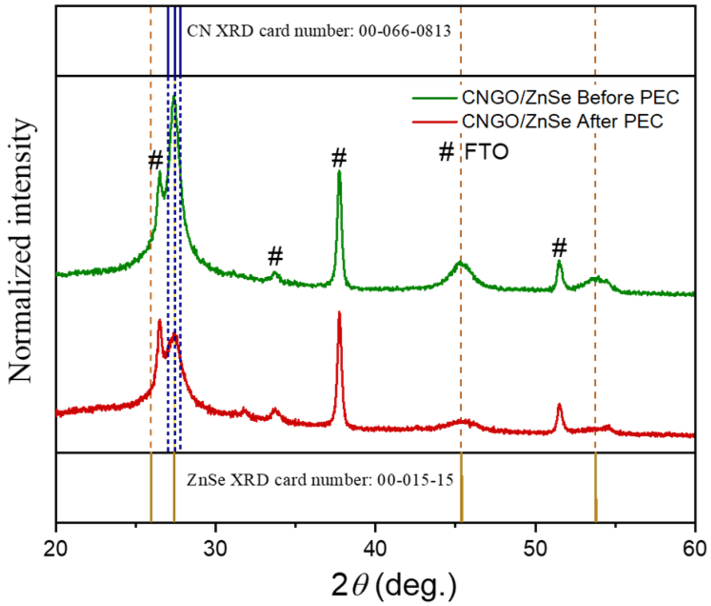

Figure S19. XRD pattern of CNGO/ZnSe film after the stability experiment. The intensities were normalized with respect to FTO's tetragonal SnO_2 (200) plane at $2\theta = 37.7°$.

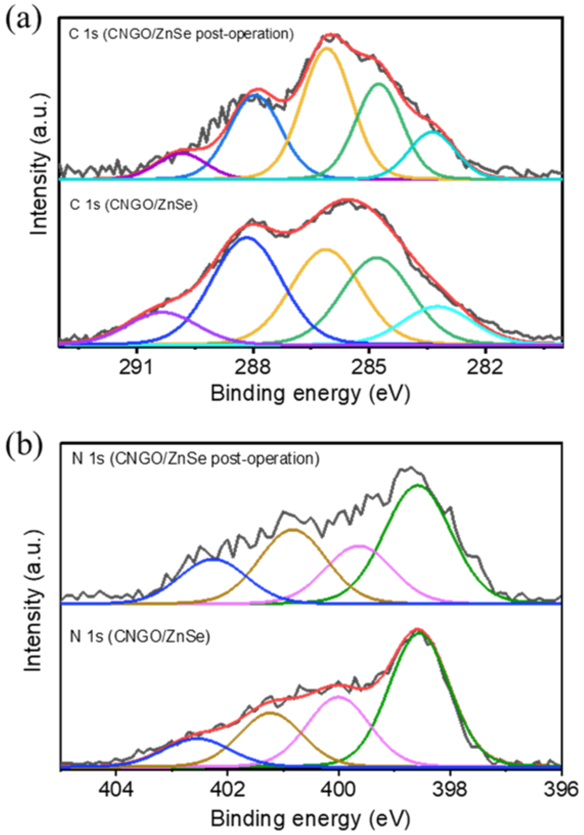

Figure S20. XPS characterization of a CNGO/ZnSe film after the stability test ('post-operation' of a 12 h water-splitting PEC experiment). (a) C 1s, and (b) N 1s spectra.

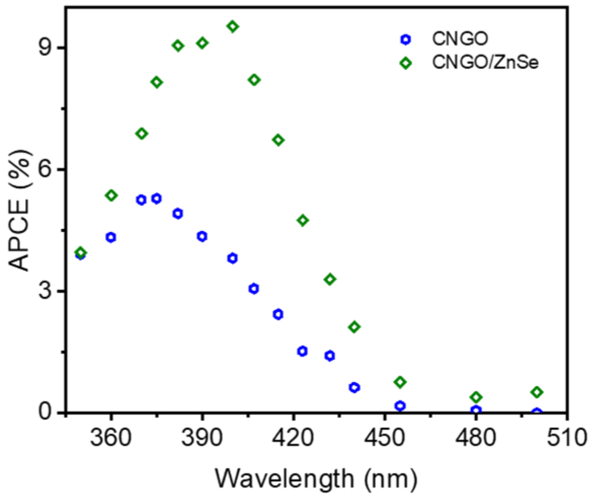

Figure S21. Absorbed photon-to-current efficiency (APCE) of CNGO and CNGO/ZnSe films.

Supporting Information References

(1) Shaviv, E.; Salant, A.; Banin, U. Size Dependence of Molar Absorption Coefficients of CdSe Semiconductor Quantum Rods. *ChemPhysChem* **2009**, *10* (7), 1028–1031. https://doi.org/https://doi.org/10.1002/cphc.200800874.

(2) Patra, P. C.; Mohapatra, Y. N. Graphitic Carbon Nitride (g-C_3N_4)/Al_2O_3 Heterostructure as Double Dielectric: A Comparative Study in MIS Based on a-IGZO. *IEEE J. Electron Devices Soc.* **2021**, *9*, 618–622.

(3) Elimelech, O.; Aviv, O.; Oded, M.; Banin, U. A Tale of Tails: Thermodynamics of CdSe Nanocrystal Surface Ligand Exchange. *Nano Lett.* **2020**, *20* (9), 6396–6403. https://doi.org/10.1021/acs.nanolett.0c01913.

(4) Yeh, C.-Y.; Lu, Z. W.; Froyen, S.; Zunger, A. Zinc-Blende--Wurtzite Polytypism in Semiconductors. *Phys. Rev. B* **1992**, *46* (16), 10086–10097. https://doi.org/10.1103/PhysRevB.46.10086.

(5) Seo, Y. J.; Das, P. K.; Arunachalam, M.; Ahn, K.-S.; Ha, J.-S.; Kang, S. H. Drawing the Distinguished Graphite Carbon Nitride (g-C3N4) on SnO2 Nanoflake Film for Solar Water Oxidation. *Int. J. Hydrogen Energy* **2020**, *45* (43), 22567–22575. https://doi.org/https://doi.org/10.1016/j.ijhydene.2020.06.143.

(6) Zhang, J.; Zou, Y.; Eickelmann, S.; Njel, C.; Heil, T.; Ronneberger, S.; Strauss, V.; Seeberger, P. H.; Savateev, A.; Loeffler, F. F. Laser-Driven Growth of Structurally Defined Transition Metal Oxide Nanocrystals on Carbon Nitride Photoelectrodes in Milliseconds. *Nat. Commun.* **2021**, *12* (1), 3224. https://doi.org/10.1038/s41467-021-23367-7.

(7) Hu, X.; Zeng, X.; Liu, Y.; Lu, J.; Yuan, S.; Yin, Y.; Hu, J.; McCarthy, D. T.; Zhang, X. Nano-Layer Based 1T-Rich MoS2/g-C3N4 Co-Catalyst System for Enhanced Photocatalytic and Photoelectrochemical Activity. *Appl. Catal. B Environ.* **2020**, *268*, 118466. https://doi.org/https://doi.org/10.1016/j.apcatb.2019.118466.

(8) Zhang, W.; Albero, J.; Xi, L.; Lange, K. M.; Garcia, H.; Wang, X.; Shalom, M. One-Pot Synthesis of Nickel-Modified Carbon Nitride Layers Toward Efficient

Photoelectrochemical Cells. *ACS Appl. Mater. Interfaces* **2017**, *9* (38), 32667–32677. https://doi.org/10.1021/acsami.7b08022.

(9) Hou, Y.; Zuo, F.; Dagg, A. P.; Liu, J.; Feng, P. Branched WO3 Nanosheet Array with Layered C3N4 Heterojunctions and CoOx Nanoparticles as a Flexible Photoanode for Efficient Photoelectrochemical Water Oxidation. *Adv. Mater.* **2014**, *26* (29), 5043–5049. https://doi.org/https://doi.org/10.1002/adma.201401032.

(10) Vinoth, S.; Ong, W.-J.; Pandikumar, A. Sulfur-Doped Graphitic Carbon Nitride Incorporated Bismuth Oxychloride/Cobalt Based Type-II Heterojunction as a Highly Stable Material for Photoelectrochemical Water Splitting. *J. Colloid Interface Sci.* **2021**, *591*, 85–95. https://doi.org/https://doi.org/10.1016/j.jcis.2021.01.104.

(11) Karimi-Nazarabad, M.; Goharshadi, E. K.; Mahdizadeh, S. J. Efficient Photoelectrocatalytic Water Oxidation by Palladium Doped G-C3N4 Electrodeposited Thin Film. *J. Phys. Chem. C* **2019**, *123* (43), 26106–26115. https://doi.org/10.1021/acs.jpcc.9b07755.

(12) Ehsan, M. F.; Fazal, A.; Hamid, S.; Arfan, M.; Khan, I.; Usman, M.; Shafiee, A.; Ashiq, M. N. CoFe2O4 Decorated G-C3N4 Nanosheets: New Insights into Superoxide Anion Mediated Photomineralization of Methylene Blue. *J. Environ. Chem. Eng.* **2020**, *8* (6), 104556. https://doi.org/https://doi.org/10.1016/j.jece.2020.104556.

(13) Liu, Y.; Zhang, J.; Li, X.; Yao, Z.; Zhou, L.; Sun, H.; Wang, S. Graphitic Carbon Nitride Decorated with CoP Nanocrystals for Enhanced Photocatalytic and Photoelectrochemical H2 Evolution. *Energy & fuels* **2019**, *33* (11), 11663–11676.

(14) Sitara, E.; Nasir, H.; Mumtaz, A.; Ehsan, M. F.; Sohail, M.; Iram, S.; Bukhari, S. A. B.; Ullah, S.; Akhtar, T.; Iqbal, A. Enhanced Photoelectrochemical Water Splitting Using Zinc Selenide/Graphitic Carbon Nitride Type-II Heterojunction Interface. *Int. J. Hydrogen Energy* **2021**, *46* (50), 25424–25435.

(15) Nunna, G. P.; Rosaiah, P.; Sangaraju, S.; Ramalingam, G.; Jwuiyad, A.; Adem, S.;

Ko, T. J. Mesostructured Graphitic Carbon Nitride Composites with Silver Nanoparticle Decoration as the Best Visible-Light-Driven Photocatalysts for Dye Degradation and H2 Production. *Colloids Surfaces A Physicochem. Eng. Asp.* **2024**, *680*, 132615.

(16) Baig, U.; Khan, A.; Gondal, M. A.; Dastageer, M. A.; Akhtar, S. Single-Step Synthesis of Silicon Carbide Anchored Graphitic Carbon Nitride Nanocomposite Photo-Catalyst for Efficient Photoelectrochemical Water Splitting under Visible-Light Irradiation. *Colloids Surfaces A Physicochem. Eng. Asp.* **2021**, *611*, 125886.
